# Concordance of Dietary Diversity and Moderation Among 28,787 Mother‐Child Dyads in 11 Low‐ and Middle‐Income Countries: Implications for Global Monitoring and Targeted Nutrition Actions

**DOI:** 10.1111/mcn.70081

**Published:** 2025-08-22

**Authors:** Giles T. Hanley‐Cook, Emma van der Meulen, Alissa M. Pries, Simone M. Gie, Nancy J. Aburto, Bridget A. Holmes

**Affiliations:** ^1^ Food and Nutrition Division Food and Agriculture Organization of the United Nations Rome Lazio Italy; ^2^ Independent consultant London UK

**Keywords:** DHS, dietary diversity, IYCF, MDD‐W, moderation

## Abstract

In 2025, the ‘Prevalence of minimum dietary diversity’ among infants and young children (IYC) aged 6–23 months and females aged 15–49 years was adopted as an additional Sustainable Development Goal 2: Zero Hunger indicator. Previous studies, mainly in high‐income countries, have reported that children's diets bear weak to moderate resemblance of their mothers' diets. Therefore, this study assessed i) the rank correlation between Minimum Dietary Diversity for Women (MDD‐W) and MDD‐IYC prevalence at country‐level and ii) the associations and concordance of nutritious and unhealthy food group consumption among mother‐child dyads using nationally representative survey data from 11 low‐ and middle‐income countries. MDD‐W was significantly higher than MDD‐IYC in each survey, but the indicators nonetheless rank correlated very strongly across countries. Discordance favoured mothers for pulses, nuts and seeds; flesh foods; vitamin A‐rich fruits and vegetables (F&V); other F&V; and fried and salty foods, while the opposite was observed for dairy products, eggs, and sweet drinks. Higher maternal dietary diversity was strongly associated with higher diversity in nutritious food group consumption among children in each country. Lastly, mothers consuming five or more out of 10 nutritious food groups—in other words, achieving MDD‐W—best discriminated whether children achieved MDD‐IYC or not. In conclusion, MDD‐IYC and MDD‐W data provide complementary insights for targeted and context‐specific food and nutrition policies and programmes, such as behavioural change and nutrition education interventions and food environment regulations, needed to improve dietary diversity and moderation of unhealthy food groups among both IYC and females of childbearing age.

## Summary

- MDD‐W and MDD‐IYC—adopted as additional SDG 2 indicators in 2025—were strongly rank‐correlated in 11 low‐ and middle‐income countries.
- Higher maternal dietary diversity was consistently associated with greater consumption of nutritious food group among infants and young children aged 6–24 months.
- Mothers were, however, more likely to consume pulses, nuts and seeds; flesh foods; vitamin A‐rich fruits and vegetables (F&V); other F&V; and fried and salty foods, while their offspring more often consumed dairy products, eggs, and sweet drinks.
- These findings support targeted and context‐specific nutrition actions that enable diverse and moderate diets for all.

## Graphical abstract

Maternal and child dietary diversity were strongly rank correlated in 11 low‐and middle‐income countries. Mothers were, however, more likely to consume pulses, nuts and seeds; flesh foods; vitamin A‐rich fruits and vegetables (F&V); other F&V; and fried and salty foods than their child, while the opposite was observed for dairy products, eggs, and sweet drinks.

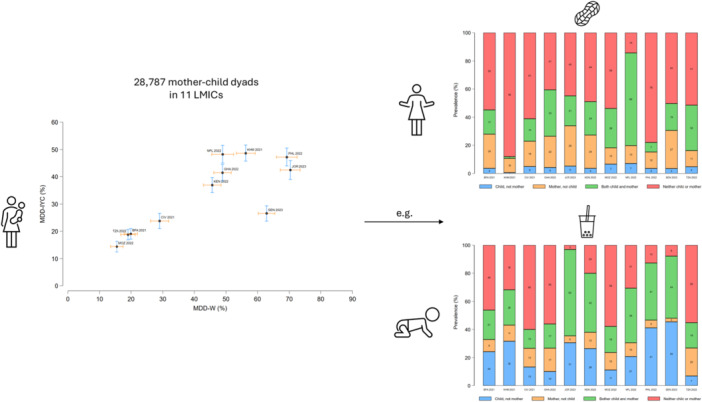

**Abbreviations**
AUC: area under the curve
CI: confidence interval
DHS: Demographic and Health Surveys
FAO: Food and Agriculture Organization of the United Nations
FGDS: food group diversity score
F&V: fruits and vegetables
IYC: infants and young children
LMIC: low‐ and middle‐income countries
LOA: limits of agreement
MDD‐IYC: Minimum Dietary Diversity for Infants and Young Children
MDD‐W: Minimum Dietary Diversity for Women
PCC: percentage correctly classified
pp: percentage points
PSU: primary sampling unit
SD: standard deviation
SDG: Sustainable Development Goals
WHO: World Health Organization
*ρ*
: Spearman's rank correlation coefficient

## 1 Introduction

Globally between 1.2 and 1.4 billion nonpregnant females (15–49 years of age) suffer from micronutrient deficiencies (Stevens et al. 2022) and over 480 million adult females are obese (Phelps et al. 2024), while more than 145 million children under‐five are stunted and at least 35 million are overweight (UNICEF et al. 2023). Females and infants and young children (IYC) are at relatively greater risk of all forms of malnutrition—in other words, undernutrition, micronutrient deficiencies, and overweight and obesity—due to the higher nutrient requirements associated with menstruation, pregnancy, and lactation for females, and growth and brain development for IYC (Allen et al. 2020). Furthermore, females and IYC also suffer from persisting inequalities in food access and cultural values (Harris‐Fry et al. 2017), often favouring excessive weight gain in many low‐ and middle‐income countries (LMICs) (Kanter and Caballero 2012).

Suboptimal dietary patterns are a common cause of all forms of malnutrition (Wells et al. 2020) and likely have detrimental short‐ and long‐term intergenerational effects, as illustrated by unhealthy diets during pregnancy being associated with a greater risk of preterm birth (Chia et al. 2019; Raghavan et al. 2019). Furthermore, the first 1000 days—the period from conception to 2 years of age—is a critical window of opportunity for ameliorating dietary patterns, as early childhood diets not only impact food preferences across the lifespan (Switkowski et al. 2020) but also effect immediate physical growth, cognitive development, gut microbiome composition, and nutritional status and health in later childhood and adulthood (Brazionis et al. 2013; Llewellyn et al. 2016; Pantazi et al. 2023).

In 2024, the Food and Agriculture Organization of the United Nations (FAO) and World Health Organization (WHO) defined a healthy diet as adequate in essential nutrients without excess, balanced in energy intake and macronutrients, moderate in foods, nutrients, or other compounds associated with detrimental health effects, and diverse in nutritious food within and across food groups (FAO and WHO 2024). At present, only food group‐based indicators, which capture dietary diversity, have been sufficiently validated for global monitoring of diets among females and IYC—namely, Minimum Dietary Diversity for Women (MDD‐W) and Minimum Dietary Diversity for Infants and Young Children (MDD‐IYC). Consuming the minimum number of food groups for each indicator has been associated with minimally acceptable levels of micronutrient adequacy across contexts (Dewey et al. 2004; Hanley‐Cook et al. 2024; Women's Dietary Diversity Project WDDP Study 2017; Verger et al. 2024).

Therefore, multi‐topic surveys, such as the Demographic and Health Surveys (DHS), and national nutrition and health surveys have integrated dietary diversity questionnaires to capture the consumption of nutritious food groups among IYC and females in over 80 countries. Additionally, a limited set of unhealthy food groups is included, due to the growing global concern over rising rates of obesity and diet‐related non‐communicable diseases, but are not used in the calculation of MDD‐W or MDD‐IYC. In view of the large evidence base supporting dietary diversity (Herforth et al. 2019; Verger et al. 2021) and the coverage of data collected worldwide,^1^ from 2025 onwards, the United Nations Statistical Commission adopted the ‘Prevalence of minimum dietary diversity, by population group (IYC aged 6–23 months and females aged 15–49 years)’ as an additional Sustainable Development Goals (SDG) 2: Zero Hunger indicator (FAO 2025).

Research, predominantly conducted in high‐income countries, has indicated that IYC's diets only weakly to moderately resemble their mothers' diets (Pervin et al. 2023). Higher maternal dietary diversity has, however, been associated with greater dietary diversity among IYC in a limited number of LMICs, although large variability was reported in the prevalence of the specific food groups being consumed (Akseer et al. 2023; Bernate Angulo et al. 2024). Owing to a plethora of socioeconomic and cultural factors and dietary preferences, IYC are regularly fed only a subset of the foods available to a household, or foods deemed appropriate for IYC, leading to certain nutritious food groups, such as animal‐source foods and fruits and vegetables (F&V), often being excluded from IYC's diets (Akseer et al. 2023; Klassen et al. 2019).

In light of the new SDG 2 indicator, and to deepen understanding of recently collected food group consumption data, this study assessed the national prevalence of minimum dietary diversity among IYC and females across 11 LMICs. Moreover, the concordance between maternal and child consumption of both nutritious and unhealthy food groups was comprehensively examined to inform targeted and country‐specific food and nutrition policies and programmes.

## 2 Methods

Our research is reported using the Strengthening the Reporting of Observational studies in Epidemiology‐nutritional epidemiology checklist (Lachat et al. 2016).

## 3 Data Sources

We used secondary cross‐sectional DHS data from survey rounds which collected food group consumption data among both females aged 15–49 years and IYC aged 6–23 months on the same day. On 9 September 2024, The DHS Programme authorized two study authors (GH‐C and EvdM) to download microdata from 11 DHS rounds, namely: Burkina Faso 2021 (INSD and ICF 2023), Cambodia 2021 (NIS et al. 2023), Côte d'Ivoire 2021 (INS and ICF 2023), Ghana 2022 (GSS and ICF 2024), Jordan 2023 (DoS and ICF 2024), Kenya 2022 (KNBS and ICF 2023), Mozambique 2022 (INE and ICF 2024), Nepal 2022 (MoHP et al. 2023), the Philippines 2022 (PSA and ICF 2023), Senegal 2023 (ANSD 2023), and Tanzania 2022 (MoH et al. 2022). There were no exclusion criteria; hence, our subsequent statistical analyses included all 199,483 females aged 15–49 years, of whom 28,787 were mothers to IYC aged 6–23 months (Table 1).

**Table 1:** Demographic and health survey round characteristics.

Country	Burkina Faso	Cambodia	Côte d'Ivoire	Ghana	Jordan	Kenya	Mozambique	Nepal	Philippines	Senegal	Tanzania
Data collection, mm/yyyy	07/2021–11/2021	09/2021–02/2022	09/2021–12/2021	10/2022–01/2023	01/2023–06/2023	02/2022–07/2022	07/2022–02/2023	01/2022–06/2022	05/2022–06/2022	01/2023–08/2023	02/2022–07/2022
Female sample size, *n* a	17,659	19,496	14,877	15,014	12,595	32,156	13,183	14,845	27,821	16,583	15,254
Mother‐child dyad sample size, *n* (weighted)^b^	3345 (3304)	2448 (2321)	2861 (2644)	2786 (2562)	2296 (2061)	2825 (2508)	2579 (2,677)	1423 (1366)	2228 (2061)	2917 (2714)	3079 (3090)

^a^ Aged 15–49 years.

^b^ Mothers aged 15–49 years and children aged 6–23 month.

### 3.1 Eight‐Point Food Group Diversity Score, the Minimum Dietary Diversity for Infants and Young Children (MDD‐IYC) Indicator, and Food Groups to Moderate for IYC

For the 8‐point food group diversity score (FGDS) for IYC, 16 nutritious food sub‐groups (i.e., variables) were aggregated into the following eight predefined food groups (WHO and UNICEF 2021): (i) breastmilk; (ii) starchy staples; (iii) pulses, nuts, and seeds; (iv) dairy products (milk, infant formula, yoghurt, and cheese); (v) flesh foods (meat, fish and seafood, poultry, and liver or other organ meats); (vi) eggs; (vii) vitamin A‐rich F&V; and (viii) other F&V. The 8‐point FGDS was constructed by summing the number of food groups consumed by the infant or young child over the previous 24‐h, as recalled by his or her mother. Following the 2017 revision of IYC feeding indicators, reaching MDD‐IYC was defined as an infant or young child consuming ≥ 5 food groups (WHO and UNICEF 2021). In addition, 10 unhealthy food sub‐groups were aggregated into three food groups (WHO and UNICEF 2021): (i) sweet foods (candies, chocolate, cakes, pastries, ice creams etc.); (ii) fried and salty foods (chips, crisps, fried dough, instant noodles, etc.); and (iii) sweet drinks (sweetened milk, sweetened yoghurt‐based, soy‐based, or nut‐based drinks, fruit juice or fruit‐flavoured drinks soda, sports or energy drinks, sweetened tea, coffee, or herbal drinks).

### 3.2 Ten‐Point Food Group Diversity Score, the Minimum Dietary Diversity for Women (MDD‐W) Indicator, and Food Groups to Moderate for Females

For the 10‐point FGDS for females aged 15–49 years, 15 food sub‐groups (i.e., variables) were aggregated into the following 10 predefined food groups (FAO 2021): (i) starchy staples; (ii) pulses; (iii) nuts and seeds; (iv) dairy products (milk, yogurt, and cheese); (v) flesh foods (meat, fish and seafood, poultry, and liver or other organ meats); (vi) eggs; (vii) dark‐green leafy vegetables; (viii) vitamin A‐rich F&V; (ix) other vegetables; and (x) other fruits. The 10‐point FGDS was constructed by summing the number of food groups recalled as being consumed by the respondent in the previous 24‐h. Reaching MDD‐W was defined as a female consuming ≥ 5 food groups, following recommendations from validation studies among nonpregnant and pregnant adolescent and adult females (Hanley‐Cook et al. 2024; Women's Dietary Diversity Project WDDP Study 2017; Verger et al. 2024). To facilitate direct comparisons with IYC, food groups ii and iii, vii and viii, and ix and x were aggregated into three broader food groups: pulses, nuts, and seeds; vitamin A‐rich F&V; and other F&V, respectively. Moreover, seven unhealthy food sub‐groups were aggregated into the following three food groups: (i) sweet foods; (ii) fried and salty foods; and (iii) sweet drinks.

### 3.3 Statistical Analysis

Data management and statistical analysis were conducted in Stata 16.1 (StataCorp LLC 2022). All analyses, for each of the 11 countries, were weighted and accounted for the stratified two‐stage cluster design (i.e., primary sampling unit (PSU) and region × residence) using the *svyset* command, while analyses of the pooled sample of 11 surveys were unweighted. Descriptive data are means and standard deviations (SDs) percentages with 95% confidence intervals (CIs).

Relationships between nationally representative MDD‐W and MDD‐IYC estimates were visualised using range plots with 95% CIs and included linear and polynomial regression predictions. In addition, we examined the Spearman's rank correlations coefficient (*ρ*) between MDD‐W and MDD‐IYC prevalence at country‐level and defined *ρ* ≥ 0.79 as very strong correlations.

For mother‐child dyads, associations between FGDS or individual food group consumption prevalence and mothers' age in years or children's age in months were visualised using kernel‐weighted polynomial regression plots with 95% CIs. We visualized concordance and discordance between mother‐child dyads using stacked bar graphs for the seven overlapping nutritious food groups (i.e., excluding breastmilk, as usually not consumed by mothers) and three unhealthy foods groups. Prevalence estimates were computed for four mutually exclusive categories: (i) food group consumed by child, but not his or her mother; (ii) food group consumed by mother, but not her child; (iii) food group consumed by both child and mother; and (iv) food group consumed by neither child nor mother. The prevalence of discordance and concordance was computed by summing categories i and ii and categories iii and iv, respectively.

To assess the concurrent positioning of mothers and children on the 10‐point and 8‐point FGDS distribution, respectively, the measurement agreement between weighted mean‐standardized FGDS was visually inspected using Bland‐Altman plots, with coordinates graphically weighted by (non‐proportional) bubbles (Martin Bland and Altman 1986; Hanley‐Cook et al. 2024). In summary, we graphed the difference between maternal and child FGDS *z*‐scores against the average of these FGDS *z*‐scores. The upper and lower limits of agreement (LOAs) were defined as mean ± 1.96 × SD.

To assess the direction and magnitude of associations between weighed mean‐standardized maternal and child FGDS by each country, we fitted ordinary least squares regression models. The normality of residuals was assessed by visual inspection of normal probability and quantile‐normal plots. If the normality assumption was violated, quantile regression models were fitted (i.e., to compare changes in medians, rather than means). To assess relationships between maternal MDD‐W and MDD‐IYC, we used both linear probability models with robust standard errors and logistic regression models.

Lastly, the MDD‐IYC prevalence by maternal 10‐point FGDS was visualised using bar graphs, and the performance of various food group cut‐offs (e.g., ≥ 4, ≥ 5, ≥ 6) in predicting MDD‐IYC was assessed using the area under the curve (AUC) from receiver operating characteristics analysis and an informed assessment of the sensitivity, specificity, and percentage correctly classified (PCC). Following previous MDD‐W validation studies, specificity was weighted slightly higher than sensitivity for cut‐off validation, as false positive findings (i.e., type I errors) were deemed less acceptable than false negative results (i.e., type II errors). Nonetheless, sensitivity, specificity, and PCC were all required to be > 60% before a maternal FGDS cut‐off was deemed valid to predict MDD‐IYC.

As robustness checks, the regression, concordance, and test characteristic analyses were repeated among young children aged 12–23 months (*n *= 18,770). Moreover, concordance and discordance of food group consumption between mother‐child dyads was stratified for breastmilk intake. Two‐sided *p* values of < 0.001 were considered significant for all statistical tests.

### 3.4 Ethical Approval

The DHS data may be used only for the purpose of statistical reporting and analysis, and only for registered research. The institutional review board‐approved procedures for DHS public‐use datasets do not in any way allow respondents, households, or sample communities to be identified. There are no names of individuals or household addresses in the data files. The geographic identifiers only go down to the regional level, where regions are typically very large geographical areas encompassing several states or provinces. Each enumeration area has a PSU number in the data file, but the PSU numbers do not have any labels to indicate their names or locations. The current study did not include any interaction or intervention with human subjects or include any access to non‐anonymized data; hence, no approval was required from an institutional review board.

## 4 Results

### 4.1 Sample Characteristics

All DHS data were collected between July 2021 and August 2023 and the length of data collection ranged between four and 8 months across the 11 countries. The sample size of females aged 15–49 years ranged between 14,877 in Côte d'Ivoire and 32,156 in Kenya, while the unweighted sample size of mother‐child dyads ranged between 1423 in Nepal and 3345 in Burkina Faso (Table 1). The weighted mean (SD) ages of mothers and children were 28.4 years (6.68) and 14.3 months (5.12), respectively.

**Figure 1:**
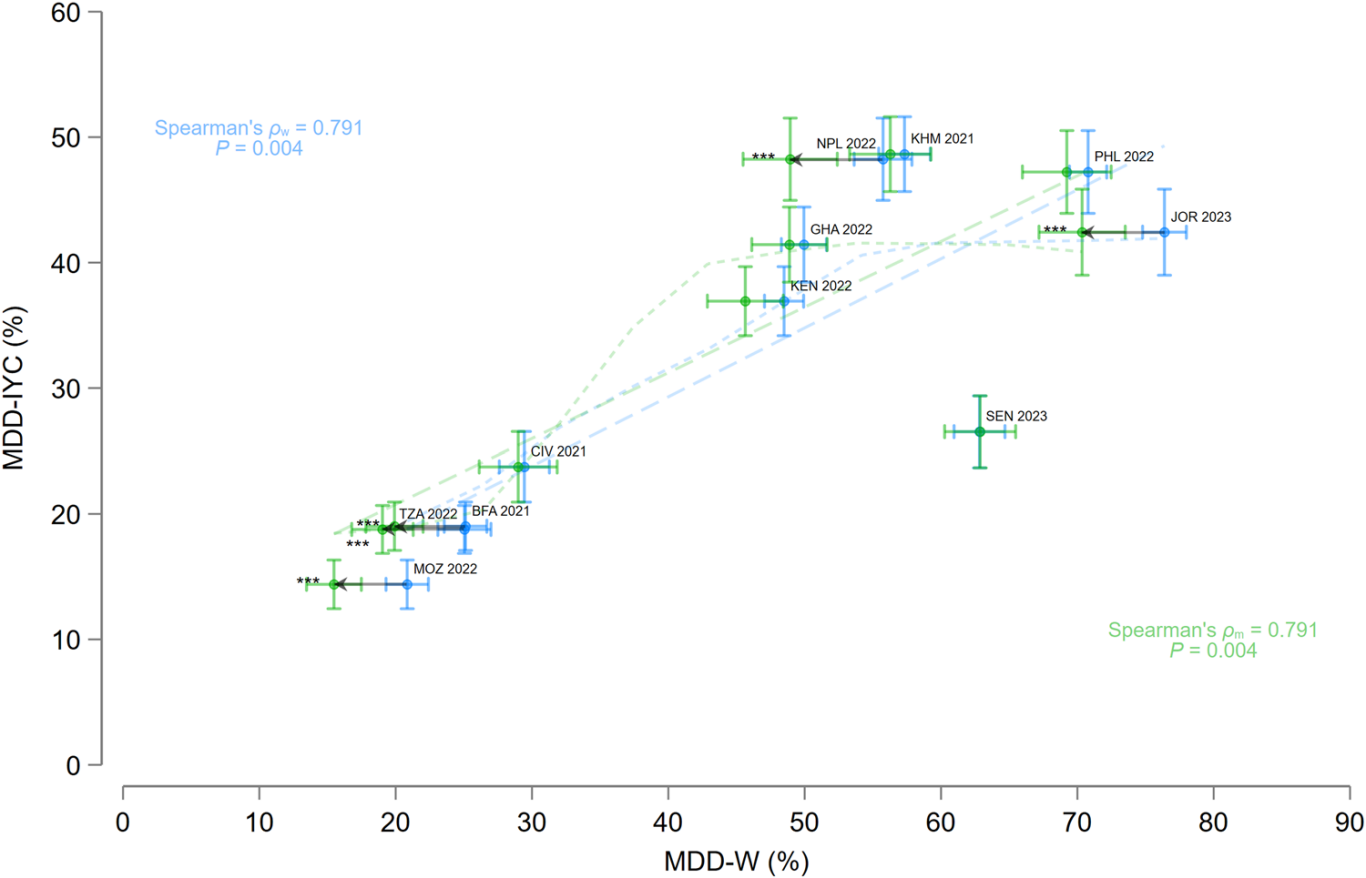
Range plot with 95% confidence intervals of Minimum Dietary Diversity for Women (MDD‐W) prevalence among females (blue) and mothers (green) aged 15–49 years and Minimum Dietary Diversity for Infants and Young Children (MDD‐IYC) aged 6–23 months, by Demographic and Healthy Survey round. The dashed lines represent the linear prediction, while the short dashed lines represent the kernel‐weighted local polynomial regression line. Arrows represent countries in which the MDD‐W prevalence among the subsample of mothers is statistically lower than among the total sample of females. *** *p* < 0.001. BFA, Burkina Faso; CIV, Republic of Côte d'Ivoire; GHA, Republic of Ghana; JOR, Hashemite Kingdom of Jordan; KEN, Republic of Kenya; KMH, Kingdom of Cambodia; MOZ, Republic of Mozambique; PHL, Republic of the Philippines; *ρ*_m_, rank correlation coefficient using sample of mothers; *ρ*_w_, rank correlation using sample of females; SEN, Republic of Senegal; TZA, United Republic of Tanzania.

The prevalence of MDD‐W (95% CI) ranged between 15.5% (13.5, 17.5) in Mozambique and 70.3% (67.2, 73.5) in Jordan (Table 2). MDD‐W prevalence was significantly lower among the subsample of mothers than among the total sample of females in Burkina Faso (95% CI: 1.6, 8.9 percentage points (pp)), Jordan (1.3, 10.8 pp), Mozambique (1.8, 8.9 pp), Nepal (1.3, 12.4 pp), and Tanzania (1.8, 10.2 pp) (Figure 1). The prevalence of MDD‐IYC (95% CI) ranged between 14.4% (12.4, 16.3) in Mozambique and 48.6% (45.7, 51.6) in Cambodia (Table 2).

**Table 2:** Nutritious food group consumption and minimum dietary diversity (MDD) prevalence (95% CI) among infants and young children (IYC) aged 6–23 months and their mothers aged 15–49 years, by demographic and health survey round^a^.

Food group	Population group	Breast milk	Starchy staples	Pulses	Nuts and seeds	Dairy products	Flesh foods	Eggs	Dark green leafy vegetables	Other vitamin A‐rich F&V	Other vegetables	Other fruits	MDD^b^
Burkina Faso 2021 (*n *= 3354)	IYC	87.1 (85.6, 88.5)	79.9 (78.3, 81.6)	15.4 (13.7, 17.2)		18.6 (16.3, 20.9)	29.8 (27.4, 32.2)	3.78 (3.03, 4.53)	53.1 (50.7, 55.6)		30.7 (28.3, 33.1)		19.0 (17.1, 21.0)
	Mothers	NA	98.3 (97.8, 98.8)	11.6 (10.0, 13.2)	35.0 (32.4, 37.6)	7.93 (6.51, 9.35)	46.9 (44.1, 49.8)	4.67 (3.66, 5.68)	74.0 (71.7, 76.4)	5.68 (4.59, 6.77)	40.5 (37.7, 43.3)	10.9 (9.19, 12.7)	19.9 (17.8, 22.0)
Cambodia 2021 (*n *= 2448)	IYC	52.6 (49.8, 55.6)	93.3 (92.0, 94.6)	2.06 (1.20, 2.91)		54.0 (50.9, 57.2)	78.1 (75.8, 80.3)	42.1 (39.4, 44.9)	55.6 (52.7, 58.4)		49.6 (46.7, 52.5)		48.6 (45.7, 51.6)
	Mothers	NA	98.8 (98.2, 99.3)	98.8 (98.2, 99.3)	9.03 (7.44, 10.6)	5.40 (3.80, 6.99)	23.7 (21.0, 26.4)	96.3 (95.3, 97.3)	41.2 (38.3, 44.0)	66.8 (64.1, 69.5)	38.1 (35.5, 40.8)	53.5 (50.8, 56.1)	50.2 (47.3, 53.0)
Côte d'Ivoire 2021 (*n *= 2861)	IYC	72.2 (69.6, 74.9)	75.2 (72.6, 77.7)	15.6 (13.6, 17.5)		29.8 (26.7, 32.8)	53.9 (50.9, 56.9)	16.4 (13.3, 19.6)	21.7 (19.3, 24.0)		46.3 (43.7, 49.0)		23.7 (20.9, 26.5)
	Mothers	NA	98.2 (97.6, 98.8)	98.2 (97.6, 98.8)	9.19 (7.82, 10.6)	27.4 (24.6, 30.2)	18.0 (15.5, 20.4)	84.1 (81.9, 86.2)	19.4 (15.7, 23.1)	26.9 (24.2, 29.7)	14.5 (12.5, 16.6)	68.6 (66.1, 71.1)	18.5 (16.0, 21.1)
Ghana 2021 (*n *= 2786)	IYC	78.1 (75.7, 80.5)	84.5 (82.7, 86.4)	28.4 (26.0, 30.7)		35.1 (32.1, 38.0)	57.1 (54.3, 59.8)	21.5 (19.0, 24.0)	31.5 (28.7, 34.2)		64.1 (61.3, 66.9)		41.4 (38.4, 44.4)
	Mothers	NA	98.8 (98.3, 99.3)	25.0 (22.9, 27.2)	36.3 (33.5, 39.1)	11.0 (9.26, 12.7)	90.9 (89.4, 92.5)	22.1 (19.6, 24.6)	74.3 (71.3, 77.3)	12.9 (10.7, 16.6)	54.3 (51.3, 57.3)	30.8 (28.2, 33.4)	48.9 (46.1, 51.6)
Jordan 2023 (*n *= 2296)	IYC	35.3 (32.5, 38.0)	81.2 (78.6, 83.8)	22.9 (20.0, 25.7)		88.9 (86.8, 91.0)	41.3 (38.1, 44.5)	45.7 (42.3, 49.1)	30.1 (27.1, 33.0)		62.5 (59.5, 65.6)		42.4 (39.0, 45.9)
	Mothers	NA	95.6 (94.3, 96.8)	32.3 (29.0, 35.6)	28.1 (24.9, 31.3)	67.1 (64.0, 70.2)	77.9 (75.3, 80.6)	48.7 (45.5, 51.9)	49.7 (46.1, 53.3)	33.5 (30.0, 37.0)	68.5 (65.2, 71.7)	62.4 (59.3, 65.4)	70.3 (67.2, 73.5)
Kenya 2022 (*n *= 2825)	IYC	79.4 (77.2, 81.6)	92.3 (91.0, 93.6)	22.1 (10.0, 25.7)		63.5 (60.8, 66.1)	21.5 (19.1, 23.9)	8.94 (7.38, 10.5)	58.3 (55.6, 60.9)		56.3 (53.4, 59.1)		36.8 (34.1, 39.6)
	Mothers	NA	98.1 (97.5, 98.7)	42.0 (39.4, 44.5)	2.73 (1.76, 3.69)	75.3 (72.8, 77.9)	35.1 (32.2, 38.0)	11.8 (9.91, 13.7)	60.5 (57.9, 63.1)	26.4 (23.9, 28.9)	49.4 (46.8, 52.1)	37.9 (35.1, 40.7)	45.6 (42.9, 48.4)
Mozambique 2022 (*n *= 2579)	IYC	77.1 (74.8, 79.4)	86.5 (84.7, 88.2)	22.1 (20.0, 24.3)		9.67 (7.86, 11.5)	32.9 (30.2, 35.6)	5.74 (4.55, 6.93)	46.7 (43.7, 49.8)		28.3 (25.7, 30.9)		14.4 (12.4, 16.3)
	Mothers	NA	97.3 (96.4, 98.3)	30.6 (27.6, 33.7)	9.52 (7.90, 11.1)	2.04 (1.42, 2.66)	48.3 (45.3, 51.3)	7.78 (6.32, 9.24)	50.0 (46.1, 53.3)	29.0 (25.8, 32.3)	32.7 (29.9, 35.5)	11.8 (10.2, 13.5)	15.5 (13.5, 17.5)
Nepal 2022 (*n *= 1423)	IYC	95.8 (94.5, 97.1)	92.2 (90.5, 93.9)	27.7 (25.1, 30.3)		47.5 (44.0, 51.0)	24.5 (21.4, 27.5)	18.4 (16.1, 20.8)	42.1 (38.7, 45.4)		48.7 (45.4, 52.1)		48.2 (45.0, 51.5)
	Mothers	NA	99.5 (99.0, 100)	79.4 (76.9, 81.8)	10.9 (8.72, 13.0)	47.2 (43.9, 50.6)	37.2 (33.7, 40.6)	14.5 (12.4, 16.7)	57.5 (53.6, 61.3)	16.8 (13.9, 19.7)	70.8 (67.7, 74.0)	31.0 (27.8, 34.1)	48.9 (45.5, 52.4)
Philippines 2022 (*n *= 2228)	IYC	63.0 (59.7, 66.4)	85.3 (82.9, 87.6)	9.40 (7.49, 11.3)		65.6 (62.5, 68.8)	49.7 (46.6, 52.7)	44.2 (40.8, 47.7)	61.1 (57.7, 64.4)		48.4 (45.2, 51.6)		47.2 (43.9, 50.5)
	Mothers	NA	99.8 (99.6, 100)	12.0 (9.80, 14.2)	12.4 (10.0, 14.8)	28.7 (25.7, 31.6)	93.4 (91.8, 95.1)	63.2 (60.3, 66.1)	60.1 (57.0, 63.3)	61.1 (57.7, 64.4)	63.0 (59.7, 66.2)	55.5 (52.1, 59.0)	69.2 (66.0, 72.5)
Senegal 2023 (*n *= 2917)	IYC	81.4 (79.6, 83.3)	72.6 (70.5, 74.8)	16.5 (14.6, 18.4)		53.7 (50.6, 56.7)	41.8 (39.1, 44.6)	8.57 (6.77, 10.4)	45.4 (42.4, 48.4)		21.3 (18.8, 23.8)		26.5 (23.7, 29.4)
	Mothers	NA	98.6 (98.1, 99.2)	26.9 (24.4, 29.4)	29.6 (26.7, 32.5)	42.5 (38.8, 46.3)	89.2 (87.5, 90.8)	12.6 (10.4, 14.7)	41.5 (38.4, 44.6)	71.1 (68.1, 74.2)	68.6 (66.0, 71.3)	18.2 (15.8, 20.5)	62.9 (60.3, 65.5)
Tanzania 2022 (*n *= 3079)	IYC	74.6 (72.2, 76.9)	94.1 (93.0, 95.2)	32.9 (30.5, 35.4)		15.4 (13.3, 17.6)	42.2 (39.3, 45.1)	5.89 (4.72, 7.06)	48.3 (45.9, 50.8)		29.7 (27.3, 32.0)		18.8 (16.9, 20.7)
	Mothers	NA	98.8 (98.4, 99.3)	43.3 (40.8, 45.8)	4.36 (3.51, 5.22)	16.9 (14.5, 19.2)	54.1 (51.1, 57.2)	2.73 (2.05, 3.40)	53.1 (50.5, 55.7)	14.3 (12.0, 16.6)	27.7 (25.2, 30.3)	20.1 (18.0, 22.2)	19.0 (16.8, 21.3)

^a^ Values are weighed estimates accounting for the stratified two‐stage cluster design. CI, confidence interval. F&V, fruits and vegetables.

^b^ MDD‐IYC is defined as an IYC consuming foods and drinks from ≥ 5 out of eight food groups, whereas MDD for Women (MDD‐W) is defined as a mother consuming foods and drinks from ≥ 5 out of 10 food groups.

Among mothers, very low nutritious food group consumption (i.e., < 20%) was reported for pulses, dairy products, eggs, other vitamin A‐rich F&V, and other fruits in Burkina Faso and Côte d'Ivoire; pulses and nuts and seeds in Cambodia and the Philippines; dairy products and other vitamin A‐rich F&V in Ghana; nuts and seeds and eggs in Kenya; nuts and seeds, dairy products, and eggs in Mozambique; nuts and seeds, eggs, and other vitamin A‐rich F&V in Nepal; eggs and other fruits in Senegal; and nuts and seeds, dairy products, eggs, and other vitamin A‐rich F&V in Tanzania (Table 2; Supporting Information S1: Table 1). Sweet food and fried and salty food consumption was high (i.e., > 30%) in Jordan, Nepal and the Philippines and sweet drinks in all countries except Ghana, Mozambique, and Tanzania (Supporting Information S1: Table 1). Mean maternal FGDS remained relatively stable across the 15–49 years age range (Supporting Information S1: Figure 1).

**Figure 2:**
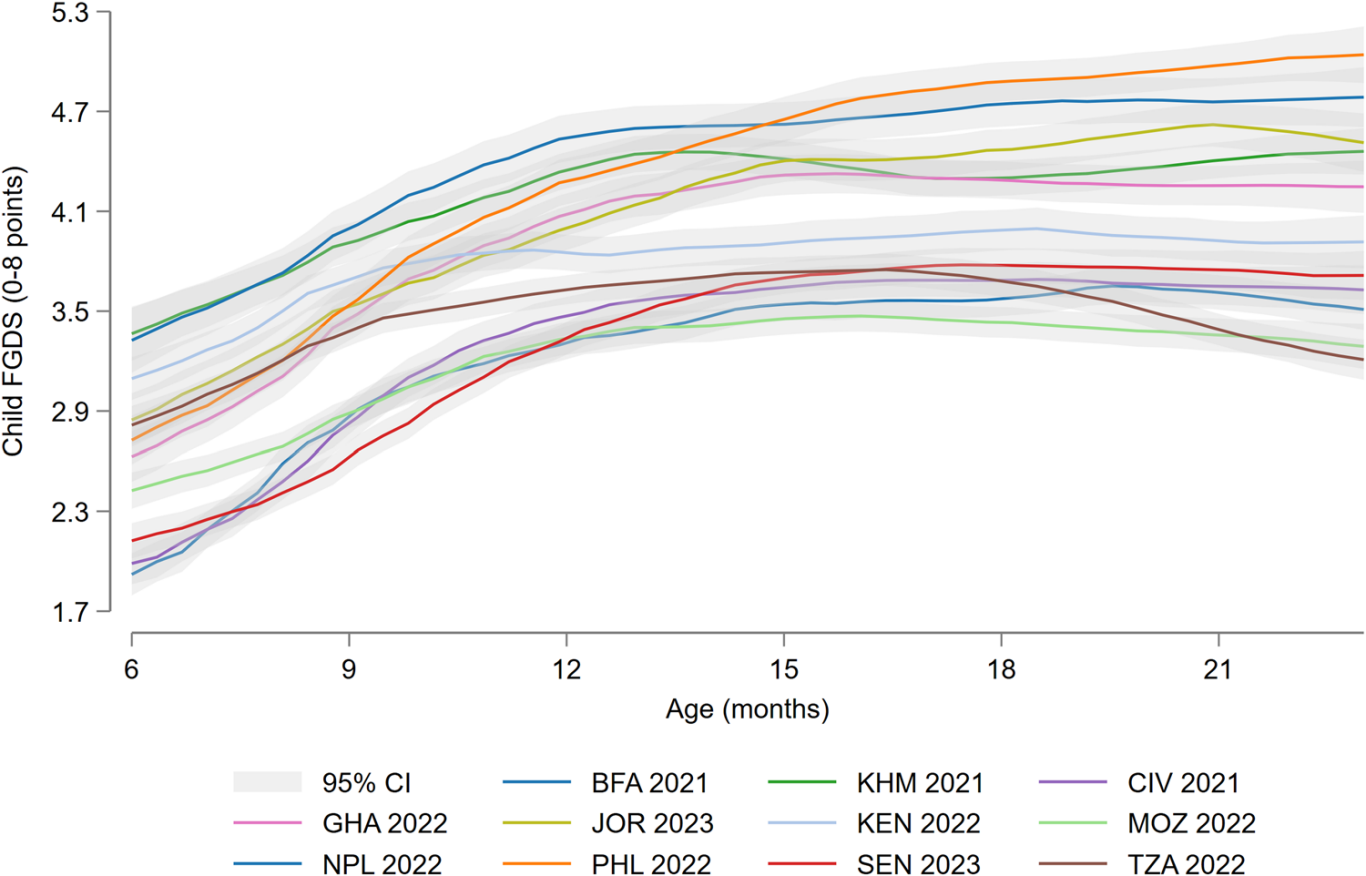
Kernel‐weighted local polynomial regression of food group diversity score (FGDS) on age among infants and young children aged 6–23 months, by Demographic and Health Surveys round. BFA, Burkina Faso; CI, confidence interval; CIV, Republic of Côte d'Ivoire; GHA, Republic of Ghana; JOR, Hashemite Kingdom of Jordan; KEN, Republic of Kenya; KMH, Kingdom of Cambodia; MOZ, Republic of Mozambique; PHL, Republic of the Philippines; SEN, Republic of Senegal; TZA, United Republic of Tanzania.

Among IYC, very low consumption was reported for the following food groups; pulses, nuts and seeds, dairy products, eggs in Burkina Faso; pulses, nuts and seeds in Cambodia and the Philippines; pulses, nuts and seeds and eggs in Côte d'Ivoire and Senegal; eggs in Kenya and Nepal; and dairy products and eggs in Mozambique and Tanzania (Table 2). Breastmilk consumption was < 40% among both IYC aged 6–23 months (Table 2) and the subsample of young children aged 12–23 months in Jordan (Supporting Information S1: Figure 2). Sweet food consumption was high in Jordan, Nepal and the Philippines, fried and salty food consumption was high in Jordan, and sweet drinks consumption in Ghana, Jordan, Kenya, Nepal, the Philippines and Senegal was also high (Supporting Information S1: Table 1).

**Figure 3:**
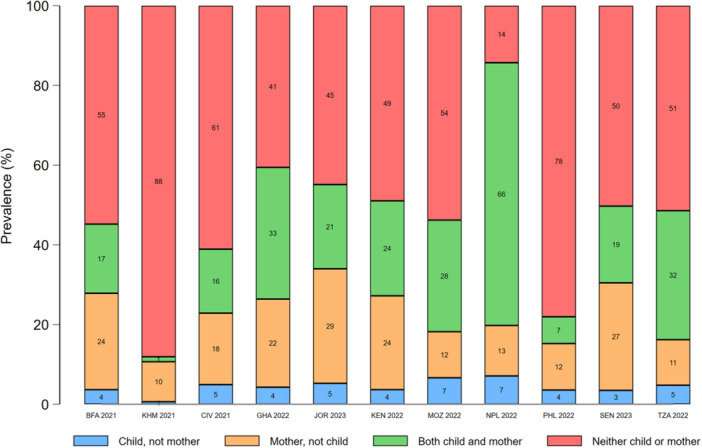
Percentage concordance and discordance between pulses, nuts and seeds consumption among infants and young children aged 12–23 months and their mothers aged 15–49 years, by Demographic and Healthy Survey round. The sum of the green and red bars is the prevalence of concordance, while the sum of the blue and orange bars is the prevalence of discordance. BFA, Burkina Faso; CIV, Republic of Côte d'Ivoire; GHA, Republic of Ghana; JOR, Hashemite Kingdom of Jordan; KEN, Republic of Kenya; KMH, Kingdom of Cambodia; MOZ, Republic of Mozambique; PHL, Republic of the Philippines; SEN, Republic of Senegal; TZA, United Republic of Tanzania.

**Figure 4:**
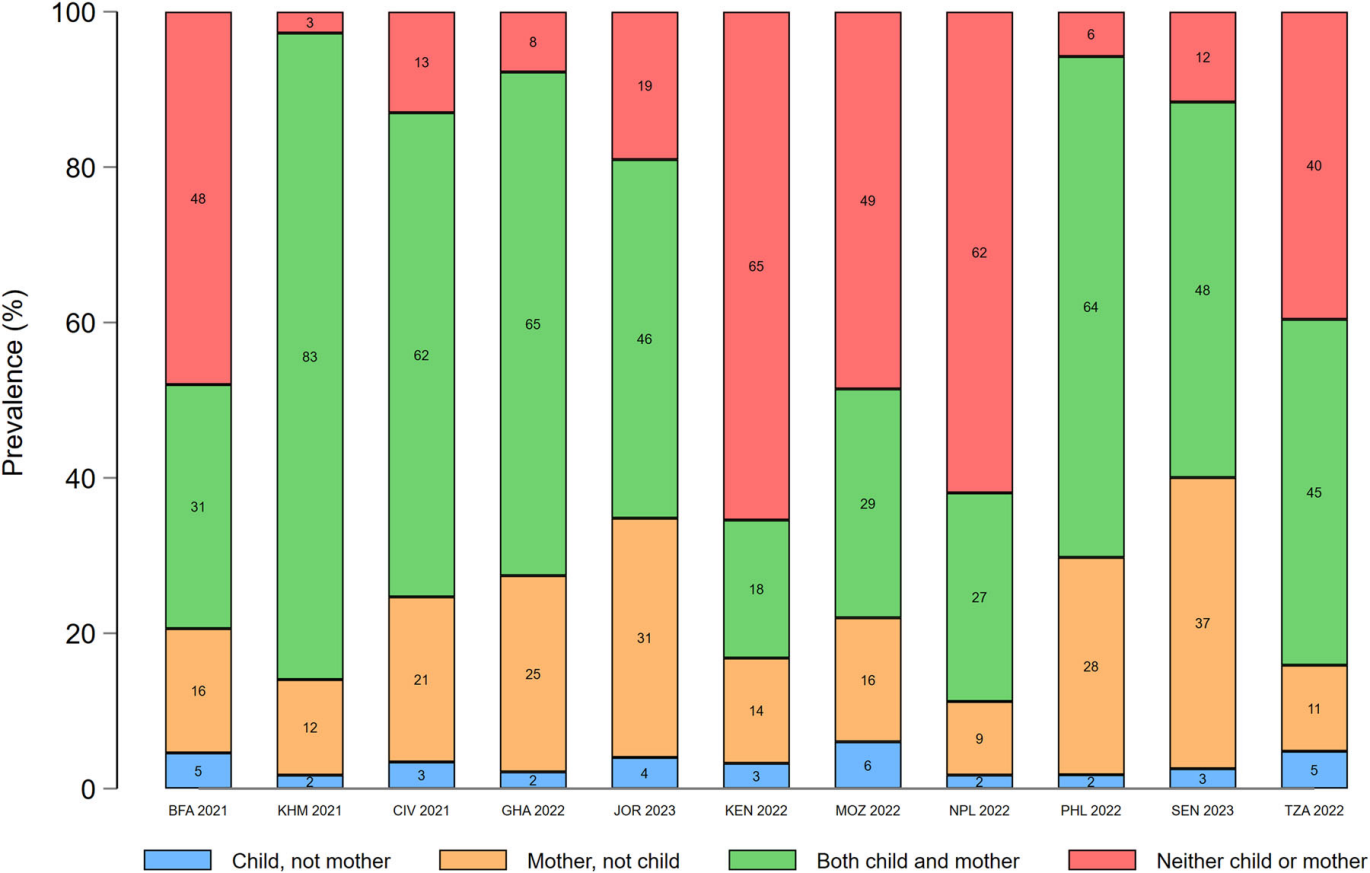
Percentage concordance and discordance between flesh foods consumption among infants and young children aged 12–23 months and their mothers aged 15–49 years, by Demographic and Healthy Survey round. The sum of the green and red bars is the prevalence of concordance, while the sum of the blue and orange bars is the prevalence of discordance. BFA, Burkina Faso; CIV, Republic of Côte d'Ivoire; GHA, Republic of Ghana; JOR, Hashemite Kingdom of Jordan; KEN, Republic of Kenya; KMH, Kingdom of Cambodia; MOZ, Republic of Mozambique; PHL, Republic of the Philippines; SEN, Republic of Senegal; TZA, United Republic of Tanzania.

Mean child FGDS increased significantly between 6 and 12 months and remains relatively stable between 12 and 23 months (Figure 2; Supporting Information S1: Table 2). The sharpest increases in food group consumption between 6 and 23 months were for starchy staples, flesh food, vitamin A‐rich F&V, other F&V, and sweet drinks, while a steep and continued decline in breastmilk consumption was observed between 6 and 23 months (Supporting Information S1: Figure 3; Supporting Information S1: Figure 4).

### 4.2 Correlation Between MDD‐W and MDD‐IYC at Country‐Level

Higher MDD‐W prevalence among all females aged 15–49 years was strongly rank correlated with MDD‐IYC prevalence among IYC aged 6–23 months. The strength of this relationship was identical among the subset of mother‐child dyads. Using maternal MDD‐W as the predictor, MDD‐IYC prevalence was higher than expected in Nepal 2022 and lower than expected in Senegal 2023 (i.e., both countries were statistical outliers) (Figure 1).

### 4.3 Measurement Agreement and Concordance Between Maternal and Child Food Group Diversity

**Figure 5:**
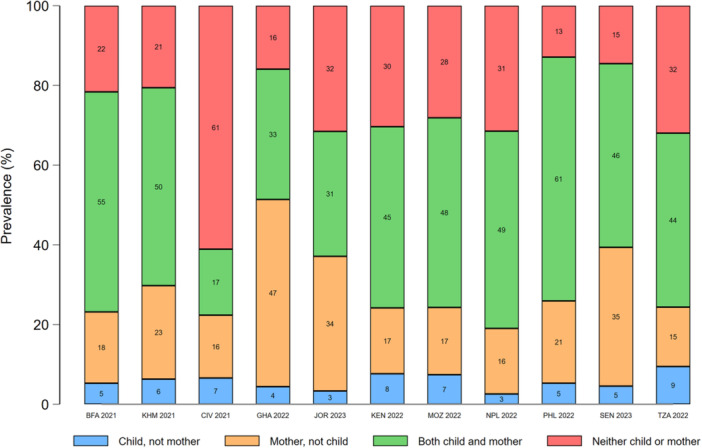
Percentage concordance and discordance between vitamin A‐rich fruits and vegetables consumption among infants and young children aged 12–23 months and their mothers aged 15–49 years, by Demographic and Healthy Survey round. The sum of the green and red bars is the prevalence of concordance, while the sum of the blue and orange bars is the prevalence of discordance. BFA, Burkina Faso; CIV, Republic of Côte d'Ivoire; GHA, Republic of Ghana; JOR, Hashemite Kingdom of Jordan; KEN, Republic of Kenya; KMH, Kingdom of Cambodia; MOZ, Republic of Mozambique; PHL, Republic of the Philippines; SEN, Republic of Senegal; TZA, United Republic of Tanzania.

**Figure 6:**
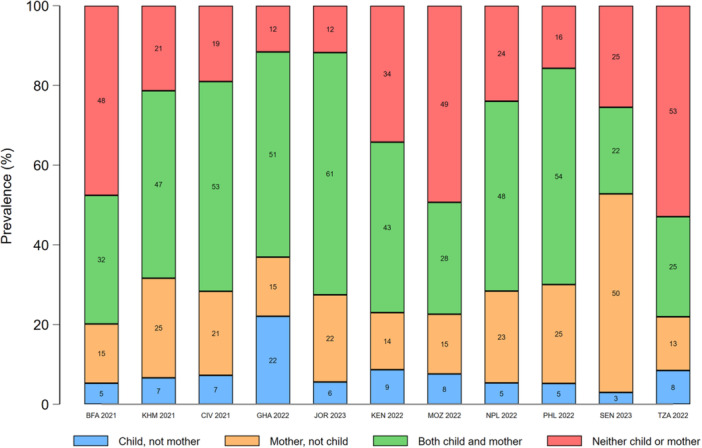
Percentage concordance and discordance between other fruits and vegetables consumption among infants and young children aged 12–23 months and their mothers aged 15–49 years, by Demographic and Healthy Survey round. The sum of the green and red bars is the prevalence of concordance, while the sum of the blue and orange bars is the prevalence of discordance. BFA, Burkina Faso; CIV, Republic of Côte d'Ivoire; GHA, Republic of Ghana; JOR, Hashemite Kingdom of Jordan; KEN, Republic of Kenya; KMH, Kingdom of Cambodia; MOZ, Republic of Mozambique; PHL, Republic of the Philippines; SEN, Republic of Senegal; TZA, United Republic of Tanzania.

The position of a mother on the 10‐point FGDS distribution (e.g., 84th percentile or +1 SD) did not always strongly agree with the position of her child on the eight‐point FGDS distribution. The LOA between standardized maternal and child FGDS was widest in Senegal (−2.32 to 2.32 *z*‐scores) and narrowest in Nepal (−1.93 to 1.77 *z*‐scores). In all countries, measurement disagreement was randomly distributed—in other words no systematic upward or downward biases were observed. Nonetheless, in Jordan and Mozambique the percentile of maternal FGDS was, on average, marginally higher (both 0.06 *z*‐scores) than the percentile of child FGDS, while in Nepal (−0.08 *z*‐scores) and the Philippines (−0.06 *z*‐scores) the direction of this relationship was reversed (Supporting Information S1: Figures 5–6).

**Figure 7:**
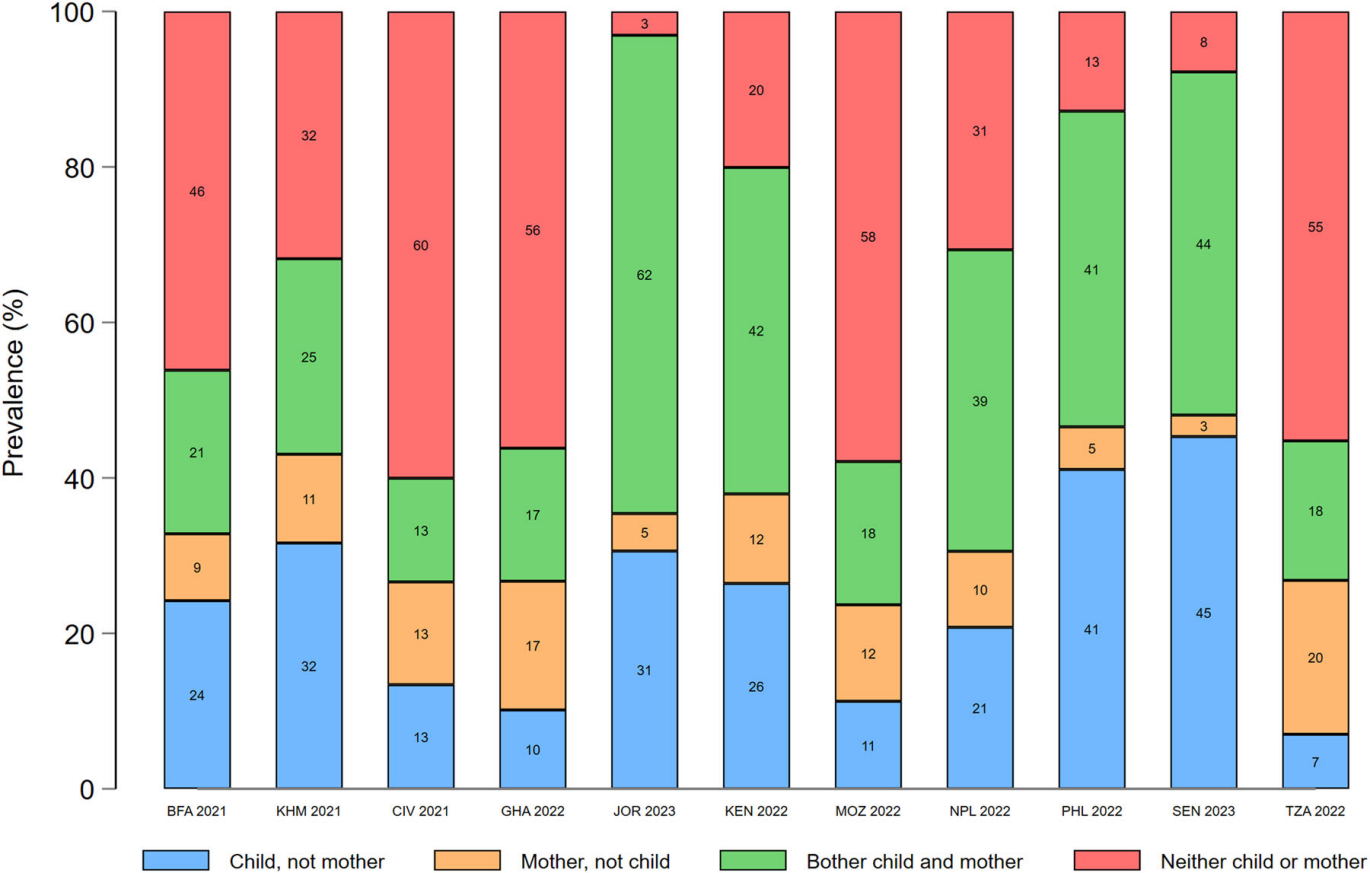
Percentage concordance and discordance between sweet drinks consumption among infants and young children aged 12–23 months and their mothers aged 15–49 years, by Demographic and Healthy Survey round. The sum of the green and red bars is the prevalence of concordance, while the sum of the blue and orange bars is the prevalence of discordance. BFA, Burkina Faso; CIV, Republic of Côte d'Ivoire; GHA, Republic of Ghana; JOR, Hashemite Kingdom of Jordan; KEN, Republic of Kenya; KMH, Kingdom of Cambodia; MOZ, Republic of Mozambique; PHL, Republic of the Philippines; SEN, Republic of Senegal; TZA, United Republic of Tanzania.

**Figure 8:**
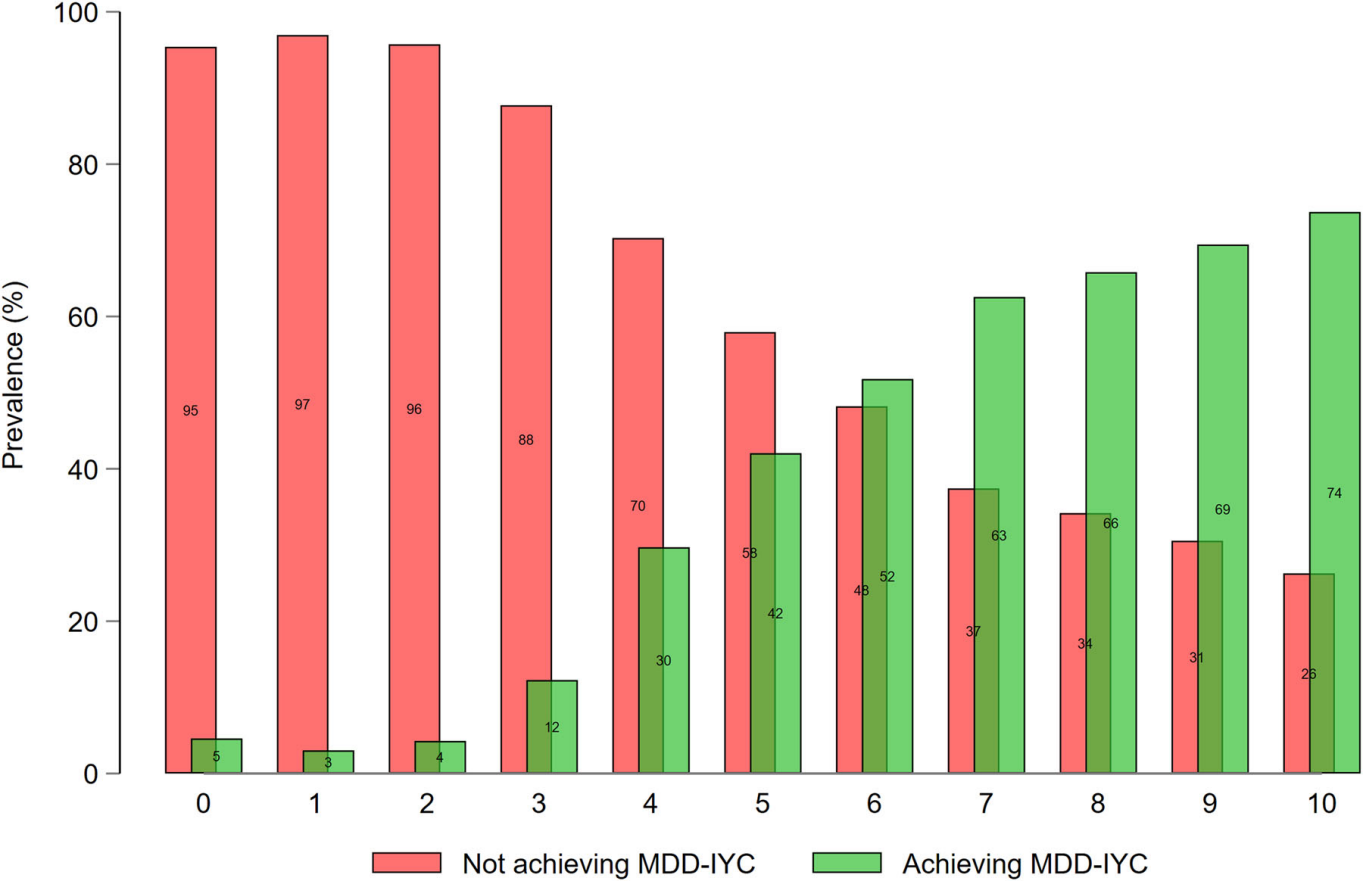
Percentage of infants and young children aged 6–23 months from 11 Demographic and Healthy Survey rounds (*n *= 28,787) achieving Minimum Dietary Diversity for Infants and Young Children (MDD‐IYC), by maternal food group diversity score (0–10 points).

**Figure 9:**
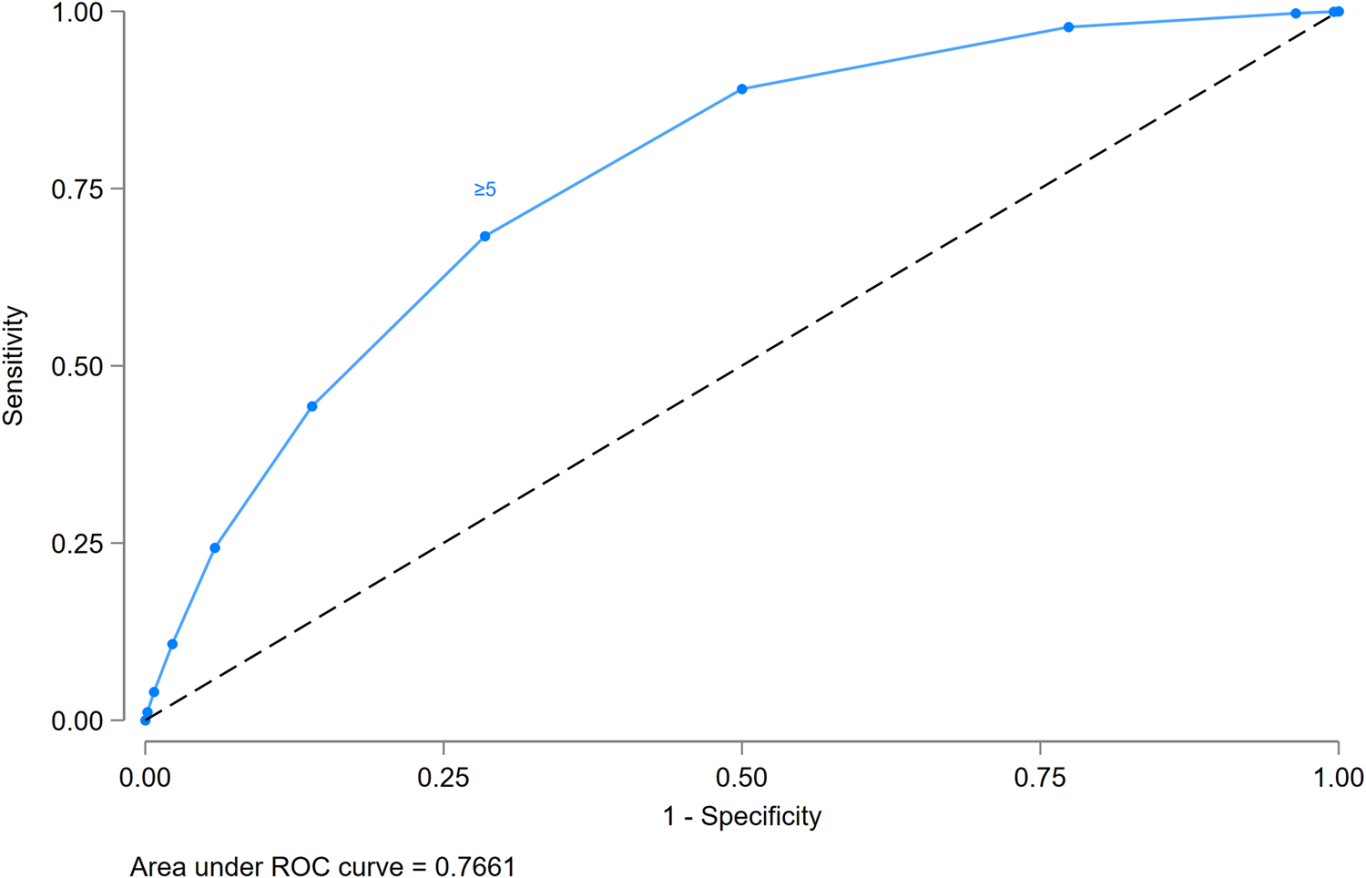
Receiver operating characteristic (ROC) curve of maternal food group diversity score (0–10 points) indicating predictions for Minimum Dietary Diversity for Infants and Young Children (MDD‐IYC) (6–23 months) from 11 Demographic and Healthy Survey rounds (*n *= 28,787). In the pooled sample, ≥ 5 food groups or Minimum Dietary Diversity for Women (MDD‐W) showed the best balance between sensitivity (86.3%), specificity (71.5%), and percentage correctly classified (70.6%).

In most countries, concordance between maternal and young child food group consumption was highest for starchy staples (range: 84%–98%) and eggs (range: 72%–95%). Discordance favoured mothers for all food groups, except dairy products, eggs, and sweet drinks (Figures 3, 4, 5, 6, 7; Supporting Information S1: Figures 7–11). Across most countries, the highest discordance was observed for pulses, nuts and seeds (maximum difference: consumed by only the mother in 29% of dyads vs. 5% only the child in Jordan) (Figure 3); flesh foods (37% vs. 3% in Senegal) (Figure 4); vitamin A‐rich F&V (47% vs. 4% in Ghana) (Figure 5); other F&V (50% vs. 3% in Senegal) (Figure 6); and sweet drinks (3% vs. 45% in Senegal) (Figure 7). Breastmilk intake did not meaningfully change the level of concordance among mother‐child dyads; nonetheless, discordance of dairy products consumption favoured non‐breastfed children even more strongly (Supporting Information S1: Figure 12).

### 4.4 Associations Between Maternal and Child Food Group Diversity

The normality assumption was met, hence untransformed data were modelled.

One‐SD increments in maternal FGDS were associated with higher child FGDS in all countries and ranged between 0.31 SD (0.26, 0.36) in Senegal and 0.55 SD (0.50, 0.60) in Kenya (Table 3). Likewise, children from mothers reaching MDD‐W were more likely to achieve MDD‐IYC in all LMICs. Differences (95% CI) in MDD‐IYC ranged between 21.7 pp (16.9, 26.6) in Senegal and 43.1 pp (38.1, 48.1) in Kenya, as compared to children from mothers not achieving MDD‐W (Table 4).

**Table 3:** Association between weighted mean‐standardized food group diversity scores (FGDS) among mothers aged 15–49 years and their infants and young children aged 6–23 months, by Demographic and Healthy Survey round^a^.

Child FGDS (*z*‐score)											
Country	Burkina faso (*n *= 3354)	Cambodia (*n *= 2448)	Côte d'Ivoire (*n *= 2861)	Ghana (*n *= 2786)	Jordan (*n *= 2296)	Kenya (*n *= 2825)	Mozambique (*n *= 2579)	Nepal (*n *= 1423)	Philippines (*n *= 2228)	Senegal (*n *= 2917)	Tanzania (*n *= 3079)
Mother FGDS (*z*‐score)	0.39 (0.34, 0.44)	0.38 (0.33, 0.42)	0.35 (0.29, 0.40)	0.35 (0.31, 0.40)	0.35 (0.29, 0.41)	0.55 (0.50, 0.60)	0.41 (0.35, 0.46)	0.51 (0.46, 0.56)	0.37 (0.31, 0.42)	0.31 (0.26, 0.36)	0.48 (0.43, 0.53)

^a^ FGDS were transformed to population group‐specific *z*‐scores using the weighted mean and weighted standard deviation (SD) within each country. Values are regression coefficients (95% confidence intervals) of a one‐SD increment in mother FGDS from ordinary least squares regression models.

**Table 4:** Association between MDD‐W among mothers aged 15–49 years and MDD‐IYC among their infants and young children aged 6–23 months, by Demographic and Healthy Survey round^a^.

	MDD‐IYC										
Country	Burkina faso (*n *= 3354)	Cambodia (*n *= 2448)	Côte d'Ivoire (*n *= 2861)	Ghana (*n *= 2786)	Jordan (*n *= 2296)	Kenya (*n *= 2825)	Mozambique (*n *= 2579)	Nepal (*n *= 1423)	Philippines (*n *= 2228)	Senegal (*n *= 2917)	Tanzania (*n *= 3079)
MDD‐W	37.9 (33.2, 42.5)	32.0 (26.8, 37.2)	31.6 (26.4, 37.8)	33.4 (28.9, 38.0)	26.7 (20.1, 33.2)	43.1 (38.1, 48.1)	32.8 (26.2, 39.4)	38.3 (33.3, 43.3)	32.1 (26.1, 38.1)	21.7 (16.9, 26.6)	34.6 (28.7, 40.5)
	7.51 (6.04, 9.33)	3.80 (3.01, 4.79)	5.01 (3.94, 6.39)	4.21 (3.40, 5.21)	3.27 (2.35, 4.55)	7.32 (6.64, 9.49)	7.09 (5.04, 9.97)	5.04 (3.98, 6.37)	3.99 (2.97, 5.35)	3.59 (2.67, 4.82)	6.34 (4.82, 9.34)

^a^ Values are percentage points and odds ratios (95% confidence intervals) from linear probability models with robust standard errors and logistic regression models, respectively. MDD‐IYC, Minimum Dietary Diversity for Infants and Young Children; MDD‐W Minimum Dietary Diversity for Women.

The magnitudes of the associations between maternal FGDS and MDD‐W with child FGDS and MDD‐IYC, respectively, were stronger among the subsample of mothers with young children aged 12–23 months (Supporting Information S1: Tables 3–4).

### 4.5 Test Characteristics and Optimal Maternal Food Group Cut‐Off for MDD‐IYC

The prevalence of MDD‐IYC increased with higher maternal FGDS (Figure 8). A maternal FGDS ≥ 5 indicated the best balance between sensitivity (86.3%), specificity (71.5%), and PCC (70.6%) for MDD‐IYC, as compared to higher or lower food group cut‐offs (Figure 9).

These findings were even stronger among the subsample of mother with young children aged 12–23 months (Supporting Information S1: Figures 13 and 14).

## 5 Discussion

Nationally representative MDD‐W prevalence, among all females or mothers only, was significantly higher than MDD‐IYC in each of the 11 countries. Nevertheless, MDD‐W and MDD‐IYC were very strongly rank‐correlated across LMICs. Furthermore, mothers of IYC had lower MDD‐W prevalence than other females aged 15–49 years, while MDD‐IYC was significantly higher and more stable among IYC aged 12–23 months, as compared to IYC aged 6–12 months. Moreover, higher maternal FGDS was strongly associated with greater child FGDS in each survey. At the population level, mothers who achieved MDD‐W best discriminated between children achieving MDD‐IYC or not. Overall, our study indicated relatively strong concordance in food group consumption between mother and child, in particular among the subsample of mothers with IYC aged 12–23 months. Nonetheless, various nutritious food groups, including pulses, nuts and seeds; flesh foods; vitamin A‐rich F&V; and other F&V, but also fried and salty foods were more likely to be consumed by mothers as compared to their children. In contrast, discordance particularly favoured IYC for dairy products and sweet drinks in the majority of countries.

Our findings are consistent with previous research in LMICs, which demonstrated strong agreement of dietary diversity among mother‐child dyads (Akseer et al. 2023; Amugsi et al. 2015; Guirindola et al. 2022; Haque et al. 2023; Kishino et al. 2023; Klassen et al. 2019; Nguyen et al. 2013; USAID 2012). In Ghana and Cambodia, between 50% and 80% of IYC whose mothers achieved minimum dietary diversity also consumed at least four out of seven foods groups—in other words, achievement of MDD‐IYC based on the prior 2008 definition (WHO 2008)—whereas less than 10% reached MDD‐IYC if their mothers consumed two or fewer food groups (USAID 2012). Another study in Ghana reported that a one food group increment in a mother's diet was associated with 0.72 food group (95% CI: 0.63, 0.82) increase in their child's diets (Amugsi et al. 2015). Furthermore, in Kenya, energy intakes and micronutrient adequacy were significantly associated among primary caregivers and children aged 2–5 years (Kishino et al. 2023).

In parallel to previous studies, starchy staples were the most commonly consumed food group and showed the highest concordance between mothers and IYC (Bernate Angulo et al. 2024). In our study, high agreement was also observed for egg consumption, especially in African countries, where very few mother‐child dyads consumed any eggs at all. In Africa, the consumption of eggs has long been a challenge due to economic constraints, but also various cultural beliefs and taboos (Maimbolwa et al. 2003). To illustrate, in various regions of Tanzania and Kenya, pregnant females reported avoiding eggs, deeming that their consumption leads to larger babies and, subsequently, birth complications (Lekey et al. 2024; Riang'a et al. 2017). Moreover, in rural regions of Tanzania, mothers reported believing that feeding eggs to IYC is a cause of baldness (USAID 2023), while in south‐eastern Nigeria, some mothers thought that egg consumption in early life predisposes children to stealing in later life (Ekwochi et al. 2016).

Our finding that discordance for vitamin A‐rich F&V consumption favoured mothers is in line with previous studies in Ghana and Cambodia (Amugsi et al. 2015; USAID 2012). Similarly, in Tanzania, mothers were more likely to consume vitamin A‐rich F&V and flesh foods as compared to their children (O'Malley et al. 2024). In contrast, a small study in Kenya indicated that children consumed more vitamin A‐rich F&V than their caregivers, although flesh food consumption was higher among their caregivers (Kishino et al. 2023). In Timor‐Leste, children were more inclined to consume dairy products and eggs than their mothers, but in contrast to our findings, also flesh foods (Bonis‐Profumo et al. 2021). However, most previous studies in LMICs such as Bangladesh, Ethiopia, Haiti, India, Indonesia, Nigeria, Tajikistan, and Viet Nam confirm our results that mother are more likely to eat many nutritious food groups than their children, in particular vitamin A‐rich F&V and flesh foods (Akseer et al. 2023; Gibson et al. 2020; Guja et al. 2021; Klassen et al. 2019; Kumar et al. 2022; Nguyen et al. 2013).

Our finding that sweet food consumption was higher among IYC than their mothers in Ghana and Kenya mirrored previous studies (Amugsi et al. 2015; Kishino et al. 2023), while our observation that discordance of sweet drink consumption favoured children in Kenya is not supported by a smaller nonrepresentative study (Kishino et al. 2023).

A myriad of socioeconomic and cultural factors, such as gender inequities, poverty, and food taboos, can impact the diets of females and IYC. To illustrate, in Tajikistan, flesh foods are deemed costly and are thus frequently not distributed to children within the household (Klassen et al. 2019). Furthermore, in Kenya and Nigeria, some studies have suggested that male household heads' control the purchasing of more expensive commodities, such as flesh foods, hampering maternal control over their own diet and those of their children (Blum et al. 2023; Bukachi et al. 2022). Moreover, many caregivers—including fathers—lack the time, knowledge, or self‐efficacy to prepare available foods for their IYC, such as grinding of flesh foods, leading to nutritious food groups being avoided until children develop teeth (Ahoya et al. 2019; Kimiywe et al. 2024; Klassen et al. 2019; Wawire 2017). Likewise, research has documented that eggs, organ meats, and pulses are sometimes avoided for IYC due to cultural beliefs and concerns such as poor digestibility, further reducing the diversity of foods provided to children (Kimiywe et al. 2024).

For evidence‐informed nutrition actions, further research is required to shed light on what factors play a role in strengthening the relationship between maternal and IYC dietary diversity. Food environments are important determinants of dietary patterns, and factors such as higher relative caloric price of nutritious food groups, as compared to starchy staples, have been negatively associated their consumption among IYC, whereas higher market assortment, or the number of different foods per food group, has been associated with greater intakes of iron‐ and vitamin‐A rich foods (Klemm et al. 2024). The extent to which these factors impact decisions on what mothers and fathers and their children eat could help to inform policy actions and interventions (Turner et al. 2020).

Furthermore, structural factors such as employment outside of the home may also contribute to differences in maternal and IYC diets. To illustrate, foods consumed away from home might contribute substantially to dietary patterns of mothers working outside of the home. If fathers are also working outside of the home, then children's diets may in turn be influenced by the choices made by secondary caregivers (e.g., grandparents) or meals and snacks provided in daycares and creches. Notably, breastmilk consumption was exceptionally low in Jordan, as compared to other LMICs in our study. This finding might be explained by challenges faced by mothers working outside of the home, such as short and unpaid maternity leave, and a general lack of social support structures for breastfeeding in Jordan (Alkhaldi et al. 2023; Al‐Sagarat et al. 2017).

At present, interventions effectively increasing nutritious food group diversity have focused predominantly on rural homestead production and behaviour change communication (e.g., cooking demonstrations, counselling) in rural areas of LMICs (Blakstad et al. 2021; Katenga‐Kaunda et al. 2021; Kuchenbecker et al. 2017; Reinbott et al. 2016; Waid et al. 2024; Waswa et al. 2015). However, a suite of multi‐sectoral food and nutrition policies and programs, such as Suaahara in Nepal (Cunningham et al. 2017), should be explored to improve dietary diversity and reduce consumption of food groups to moderate across population groups and the urban‐rural continuum (Hawkes et al. 2020; UNICEF 2022). These nutrition actions might include fiscal measures (e.g. subsidies for health protective food groups) (Comini et al. 2024), social transfers including cash and food vouchers, food labelling, food advertisement and marketing restrictions (e.g., breastmilk substitutes in Ghana (Laar et al. 2020), workplace and daycare meal procurement plans, and awareness campaigns (e.g., “Golden 1000 Days” in Nepal (Karn et al. 2017)).

Our study has several key strengths. First, the large, recently collected, and heterogeneous sample from 11 LMICs improves the generalisability our findings. Second, dietary assessment was conducted on the same day for mothers and their child allowing for direct comparison of dietary diversity. Third, as MDD‐W and MDD‐IYC are not measured on the same scale, weighted mean‐standardized agreement analysis of maternal and child FGDS was employed. Fourth, our study identified that the prevalence of mothers achieving MDD‐W is a valid indicator for whether their children reached MDD‐IYC at the population level. However, future studies might leverage the uptick in nationally‐representative, high‐frequency data collection among females to assess whether changes in MDD‐W (i.e., tipping points) can be used as an early warning for changes in MDD‐IYC. Fifth, we extended previous concordance analyses to include food groups to moderate, such as sweet foods, fried and salty foods, and sweet drinks.

Nevertheless, our analyses are also subject to limitations. Self‐reported dietary assessments are prone to recall biases and, in particular, dietary diversity of IYC might be misreported as mothers may not be solely responsible for feeding their child. Moreover, single day dietary intake assessments fail to reflect usual intakes of mother‐child dyads; hence, disagreement of episodically consumed food groups is likely overestimated. Furthermore, the rigour of the food list development or their item equivalence for females and IYC within and across DHS rounds could not be examined. In addition, data collection for DHS does not cover an entire year, and maternal and child dietary diversity has been shown to vary across seasons in multiple LMICs (Hanley‐Cook et al. 2022; Thorne‐Lyman et al. 2021). Lastly, while maternal dietary diversity has consistently been shown to be the most important predictor of children's dietary diversity (Bonis‐Profumo et al. 2021; Gibson et al. 2020; Nguyen et al. 2013), our analyses were not stratified for other potential determinants of the observed discordance among mother‐child dyads, such as educational attainment or residency (e.g., urban or rural) (Akseer et al. 2023).

In conclusion, while dietary diversity is strongly linked among mother‐child dyads, the maternal consumption of various nutritious food groups is not always predictive of consumption by their IYC, or vice versa, and unhealthy food group consumption showed poor agreement. Therefore, global monitoring of dietary intake among both females and IYC, as envisioned for SDG 2: Zero Hunger, will provide the complementary dietary data needed to inform targeted and context‐specific food and nutrition policies and programmes aimed at improving habitually low dietary diversity and rising consumption of food groups to moderate in these population groups.

## Author Contributions

The authors' responsibilities were as follows: **Giles T. Hanley‐Cook:** designed research; **Giles T. Hanley‐Cook** and **Emma van der Meulen:** analysed data and performed statistical analysis; **Giles T. Hanley‐Cook** and **Emma van der Meulen:** curated and managed data; **Giles T. Hanley‐Cook**, **Emma van der Meulen,** and **Simone M. Gie:** wrote the paper; **Giles T. Hanley‐Cook** and **Bridget A. Holmes:** have primary responsibility for final content; and all authors: read, edited and approved the final manuscript.

## Disclosure

The views expressed in this publication are those of the authors and do not necessarily reflect the views or policies of the Food and Agriculture Organization of the United Nations.

## Conflicts of Interest

The authors declare no conflicts of interest.

## Supporting information


**Supplemental Figure 1:** Kernel‐weighted local polynomial regression of food group diversity score (FGDS) on age among mothers aged 15‐49 years, by Demographic and Health Surveys round. **Supplemental Figure 2:** Percentage of infants and young children aged 12‐23 months consuming breast milk, by Demographic and Healthy Survey round. **Supplemental Figure 3:** Kernel‐weighted local polynomial regression of nutritious food group consumption prevalence on age among infants and young children aged 6‐23 months from 11 Demographic and Health Surveys round. **Supplemental Figure 4:** Kernel‐weighted local polynomial regression of unhealthy food group consumption prevalence on age among infants and young children aged 6‐23 months from 11 Demographic and Health Surveys (DHS) round. **Supplemental Figure 5:** Bland‐Altman plots of weighted mean‐standardized food group diversity score (FGDS) among infants and young children aged 6‐23 months and their mothers aged 15‐49 years, by Demographic and Healthy Survey round. **Supplemental Figure 6:** Bland‐Altman plot of weighted mean‐standardized food group diversity score (FGDS) among infants and young children aged 6‐23 months and their mothers aged 15‐49 years, by Demographic and Healthy Survey round. **Supplemental Figure 7:** Percentage concordance and discordance between starchy staples consumption among infants and young children aged 12‐23 months and their mothers aged 15‐49 years, by Demographic and Healthy Survey round. **Supplemental Figure 8:** Percentage concordance and discordance between dairy product consumption among infants and young children aged 12‐23 months and their mothers aged 15‐49 years, by Demographic and Healthy Survey round. **Supplemental Figure 9:** Percentage concordance and discordance between eggs consumption among infants and young children aged 12‐23 months and their mothers aged 15‐49 years, by Demographic and Healthy Survey round. **Supplemental Figure 10:** Percentage concordance and discordance between sweet foods consumption among infants and young children aged 12‐23 months and their mothers aged 15‐49 years, by Demographic and Healthy Survey round. **Supplemental Figure 11:** Percentage concordance and discordance between fried and salty foods consumption among infants and young children aged 12‐23 months and their mothers aged 15‐49 years, by Demographic and Healthy Survey round. **Supplemental Figure 12:** Percentage concordance and discordance between dairy products consumption among breastfed and non‐breastfed infants and young children aged 12‐23 months and their mothers aged 15‐49 years, by Demographic and Healthy Survey round. **Supplemental Figure 13:** Percentage of infants and young children aged 12‐23 months from 11 Demographic and Healthy Survey rounds (*n* = 18,770) achieving Minimum Dietary Diversity for Infants and Young Children (MDD‐IYC), by maternal food group diversity score (0‐10 points). **Supplemental Figure 14:** Supplemental figure 14. Receiver operating characteristic (ROC) curve of maternal food group diversity score (0‐10 points) indicating predictions for Minimum Dietary Diversity for Infants and Young Children (MDD‐IYC) (12‐23 months) from 11 Demographic and Healthy Survey rounds (*n* = 18,770). **Supplemental Table 1:** Unhealthy food group consumption prevalence (95% CI) among infants and young children aged 6‐23 months and their mothers aged 15‐49 years, by Demographic and Healthy Survey round^†^. **Supplemental Table 2:** Minimum Dietary Diversity prevalence (95% CI) among infants and young children aged 12‐23 months and their mothers aged 15‐49 years, by Demographic and Healthy Survey round^†^. **Supplemental Table 3:** Association between weighted mean‐standardized food group diversity scores (FGDS) among mothers aged 15‐49 years and their infants and young children aged 12‐23 months, by Demographic and Healthy Survey round†. **Supplemental Table 4:** Association between MDD‐W among mothers aged 15‐49 years and MDD‐IYC among their infants and young children aged 12‐23 months, by Demographic and Healthy Survey round.

## Data Availability

The data that support the findings of this study are available from Demographic and Health Survey Programme. Restrictions apply to the availability of these data, which were used under license for this study. Data are available from: https://dhsprogram.com/data/available-datasets.cfm with the permission of Demographic and Health Survey Programme. All data supporting the reported results are publicly available as a registered user on the Demographic and Health Survey website: https://dhsprogram.com/data/available-datasets.cfm.

## Endnotes

1 For the 2025 Comprehensive Review, the Inter Agency and Expert Group on Sustainable Development Goals set the following specific criteria (UNSD 2024): “Data must be available for at least 40% of United Nations Member States and 40% of the population across the different regions where the indicator is relevant.”
