# Supplementary material for: Concordance of Dietary Diversity and Moderation Among 28,787 Mother‐Child Dyads in 11 Low‐ and Middle‐Income Countries: Implications for Global Monitoring and Targeted Nutrition Actions
Source: Matern Child Nutr. 2025 Aug 22;22(1):e70081. doi: 10.1111/mcn.70081 (PMC12893518; doi:10.1111/mcn.70081)
Supplement: Supplementary file 1 — Supplemental Figure 1: Kernel‐weighted local polynomial regression of food group diversity score (FGDS) on age among mothers aged 15‐49 years, by Demographic and Health Surveys round. Supplemental Figure 2: Percentage of infants and young children aged 12‐23 months consuming breast milk, by Demographic and Healthy Survey round. Supplemental Figure 3: Kernel‐weighted local polynomial regression of nutritious food group consumption prevalence on age among infants and young children aged 6‐23 months from 11 Demographic and Health Surveys round. Supplemental Figure 4: Kernel‐weighted local polynomial regression of unhealthy food group consumption prevalence on age among infants and young children aged 6‐23 months from 11 Demographic and Health Surveys (DHS) round. Supplemental Figure 5: Bland‐Altman plots of weighted mean‐standardized food group diversity score (FGDS) among infants and young children aged 6‐23 months and their mothers aged 15‐49 years, by Demographic and Healthy Survey round. Supplemental Figure 6: Bland‐Altman plot of weighted mean‐standardized food group diversity score (FGDS) among infants and young children aged 6‐23 months and their mothers aged 15‐49 years, by Demographic and Healthy Survey round. Supplemental Figure 7: Percentage concordance and discordance between starchy staples consumption among infants and young children aged 12‐23 months and their mothers aged 15‐49 years, by Demographic and Healthy Survey round. Supplemental Figure 8: Percentage concordance and discordance between dairy product consumption among infants and young children aged 12‐23 months and their mothers aged 15‐49 years, by Demographic and Healthy Survey round. Supplemental Figure 9: Percentage concordance and discordance between eggs consumption among infants and young children aged 12‐23 months and their mothers aged 15‐49 years, by Demographic and Healthy Survey round. Supplemental Figure 10: Percentage concordance and discordance between sweet foods consumption amon [file MCN-22-e70081-s001.docx]

**Supplemental table 1. Unhealthy food group consumption prevalence (95% CI) among infants and young children aged 6-23 months and their mothers aged 15-49 years, by Demographic and Healthy Survey round^†^**

| **Population group** | **Children** | | | **Mothers** | | |
| --- | --- | --- | --- | --- | --- | --- |
| **Food group** | **Sweet foods** | **Fried and salty foods** | **Sweet drinks** | **Sweet foods** | **Fried and salty foods** | **Sweet drinks** |
| **Burkina Faso 2021**  **(*n*=3,354)** | 13.4  (11.8, 14.9) | 3.28  (2.65, 3.91) | 22.9  (20.8, 25.1) | 14.4  (12.8, 16.0) | 6.16  (5.08, 7.23) | 42.0  (39.1, 44.9) |
| **Cambodia 2021**  **(*n*=2,448)** | 11.8  (10.2, 13.4) | 6.22  (5.18, 7.27) | 28.4  (26.1, 30.8) | 18.0  (15.8, 20.2) | 14.7  (12.7, 16.6) | 61.5  (58.9, 64.1) |
| **Côte d’Ivoire 2021**  **(*n*=2,861)** | 10.5  (7.97, 13.0) | 8.10  (6.51, 9.68) | 26.1  (23.4, 28.8) | 13.1  (11.3, 14.9) | 16.0  (13.9, 18.2) | 30.0  (26.8, 33.3) |
| **Ghana 2021**  **(*n*=2,786)** | 20.9  (18.8, 23.1) | 3.94  (2.99, 4.89) | 31.6  (28.9, 34.3) | 17.4  (15.2, 19.6) | 6.76  (5.35, 8.17) | 28.7  (25.9, 31.4) |
| **Jordan 2023**  **(*n*=2,296)** | 32.9  (30.3, 35.4) | 33.3  (30.6, 35.9) | 56.3  (53.3, 59.4) | 68.9  (65.6, 72.3) | 48.0  (44.5, 51.4) | 92.3  (90.6, 94.0) |
| **Kenya 2022**  **(*n*=2,825)** | 12.3  (10.8, 13.8) | 19.5  (17.7, 21.2) | 49.1  (46.6, 51.6) | 11.3  (9.63, 13.1) | 22.0  (19.6, 24.4) | 69.1  (66.7, 71.4) |
| **Mozambique 2022**  **(*n*=2,579)** | 5.74  (4.69, 6.79) | 3.54  (2.66, 4.41) | 20.4  (18.1, 22.8) | 5.70  (4.62, 6.77) | 5.23  (4.03, 6.44) | 21.7  (18.9, 24.4) |
| **Nepal 2022**  **(*n*=1,423)** | 45.3  (42.6, 47.9) | 27.2  (24.6, 29.8) | 43.3  (40.1, 46.5) | 35.4  (32.2, 38.5) | 30.8  (28.2, 33.4) | 60.3  (56.7, 63.8) |
| **Philippines 2022**  **(*n*=2,228)** | 32.1  (29.3, 34.9) | 9.19  (7.69, 10.7) | 34.6  (31.4, 37.8) | 53.8  (50.4, 57.2) | 32.3  (29.1, 35.4) | 84.4  (82.1, 86.7) |
| **Senegal 2023**  **(*n*=2,917)** | 18.2  (16.2, 20.0) | 24.4  (22.1, 26.6) | 43.6  (40.7, 46.4) | 17.6  (15.3, 20.0) | 13.8  (12.0, 15.6) | 89.9  (88.3, 91.4) |
| **Tanzania 2022**  **(*n*=3,079)** | 2.86  (2.28, 3.45) | 3.45  (2.71, 4.20) | 30.0  (27.5, 32.4) | 2.82  (2.12, 3.52) | 8.72  (7.30, 10.1) | 22.0  (19.7, 24.3) |

^†^Vales are weighed estimates accounting for the stratified two-stage cluster design. CI, confidence interval.

**Supplemental table 2. Minimum Dietary Diversity prevalence (95% CI) among infants and young children aged 12-23 months and their mothers aged 15-49 years, by Demographic and Healthy Survey round^†^**

| **Food group** | **MDD-IYC** | **MDD-W** |
| --- | --- | --- |
| **Burkina Faso 2021**  **(*n*=2,219)** | 22.4  (19.9, 24.8) | 20.2  (17.7, 22.7) |
| **Cambodia 2021**  **(*n*=1,603)** | 54.0  (50.5, 57.5) | 57.4  (54.0, 60.9) |
| **Côte d’Ivoire 2021**  **(*n*=1,807)** | 29.2  (25.6, 32.8) | 31.2  (27.7, 35.0) |
| **Ghana 2021**  **(*n*=1,853)** | 47.7  (44.1, 51.3) | 49.7  (46.5, 53.0) |
| **Jordan 2023**  **(*n*=1,471)** | 52.7  (48.3, 57.1) | 70.2  (66.4, 73.8) |
| **Kenya 2022**  **(*n*=1,779)** | 40.8  (37.5, 44.2) | 45.4  (42.1, 48.7) |
| **Mozambique 2022**  **(*n*=1,660)** | 17.3  (14.8, 19.7) | 16.7  (14.3, 19.1) |
| **Nepal 2022**  **(*n*=972)** | 55.6  (51.8, 59.4) | 49.4  (45.5, 53.3) |
| **Philippines 2022**  **(*n*=1,458)** | 59.3  (55.1, 63.5) | 69.3  (65.4, 73.2) |
| **Senegal 2023**  **(*n*=1,953)** | 34.4  (30.6, 38.2) | 63.3  (60.1, 66.6) |
| **Tanzania 2022**  **(*n*=1,995)** | 21.0  (18.5, 23.6) | 18.8  (16.3, 21.3) |

^†^Vales are weighed estimates accounting for the stratified two-stage cluster design. CI, confidence interval; F&V, fruits and vegetables; MDD-IYC, Minimum Dietary Diversity for Infants and Young Children; MDD-W, Minimum Dietary Diversity for Women.

**Supplemental table 3. Association between weighted mean-standardized food group diversity scores (FGDS) among mothers aged 15-49 years and their infants and young children aged 12-23 months, by Demographic and Healthy Survey round^†^**

|  | **Child FGDS (*z*-score)** | | | | | | | | | | |
| --- | --- | --- | --- | --- | --- | --- | --- | --- | --- | --- | --- |
| **Country, *n*** | **Burkina Faso**  **(*n*=2,219)** | **Cambodia**  **(*n*=1,603)** | **Côte d’Ivoire**  **(*n*=1,807)** | **Ghana**  **(*n*=1,853)** | **Jordan**  **(*n*=1,471)** | **Kenya**  **(*n*=1,779)** | **Mozambique**  **(*n*=1,660)** | **Nepal**  **(*n*=972)** | **Philippines**  **(*n*=1,458)** | **Senegal**  **(*n*=1,953)** | **Tanzania**  **(*n*=1,995)** |
| **Mother FGDS (*z*-score)** | 0.45  (0.41, 0.50) | 0.38  (0.33, 0.43) | 0.40  (0.34, 0.45) | 0.37  (0.33, 0.42) | 0.39  (0.32, 0.45) | 0.60  (0.56, 0.66) | 0.40  (0.34, 0.47) | 0.55  (0.49, 0.60) | 0.41  (0.36, 0.47) | 0.33  (0.28, 0.38) | 0.52  (0.48, 0.58) |

^†^FGDS were transformed to population group-specific *z*-scores using the weighted mean and weighted standard deviation (SD) within each country. Values are regression coefficients (95% confidence intervals) of a one-SD increment in mother FGDS from ordinary least squares regression models.

**Supplemental table 4. Association between MDD-W among mothers aged 15-49 years and MDD-IYC among their infants and young children aged 12-23 months, by Demographic and Healthy Survey round^†^**

|  | **MDD-IYC** | | | | | | | | | | |
| --- | --- | --- | --- | --- | --- | --- | --- | --- | --- | --- | --- |
| **Country, *n*** | **Burkina Faso**  **(*n*=2,219)** | **Cambodia**  **(*n*=1,603)** | **Côte d’Ivoire**  **(*n*=1,807)** | **Ghana**  **(*n*=1,853)** | **Jordan**  **(*n*=1,471)** | **Kenya**  **(*n*=1,779)** | **Mozambique**  **(*n*=1,660)** | **Nepal**  **(*n*=972)** | **Philippines**  **(*n*=1,458)** | **Senegal**  **(*n*=1,953)** | **Tanzania**  **(*n*=1,995)** |
| **MDD-W** | 45.4  (40.1, 50.7)  9.31  (7.26, 11.9) | 35.8  (29.3, 42.2)  4.47  (3.32, 6.02) | 37.9  (31.5, 44.2)  5.88  (4.39, 7.86) | 37.0  (31.3, 42.7)  4.74  (3.65, 6.18) | 34.6  (26.3, 42.9)  4.29  (2.90, 6.34) | 45.5  (39.0, 52.1)  7.59  (5.44, 10.6) | 31.1  (23.1, 39.0)  5.52  (3.72, 8.18) | 42.5  (36.3, 48.7)  6.38  (4.68, 8.69) | 39.2  (31.6, 46.7)  5.25  (3.69, 7.47) | 26.7  (20.8, 32.6)  3.74  (2.74, 5.10) | 30.8  (30.8, 44.2)  6.52  (4.74, 8.96) |

^†^Values are percentage points and odds ratios (95% confidence intervals) from linear probability models with robust standard errors and logistic regression models, respectively. MDD-IYC, Minimum Dietary Diversity for Infants and Young Children; MDD-W Minimum Dietary Diversity for Women.

**
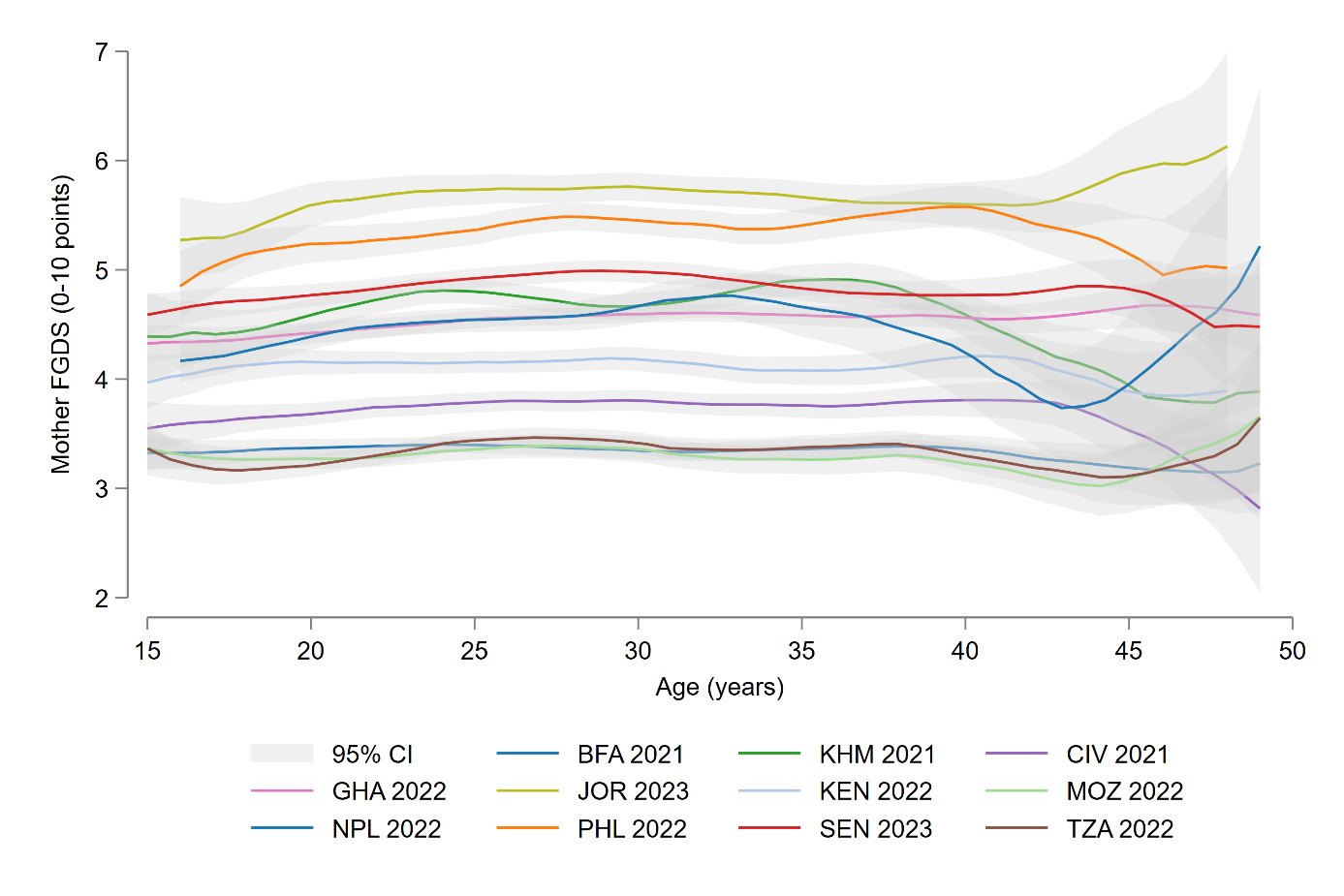
Supplemental figure 1. Kernel-weighted local polynomial regression of food group diversity score (FGDS) on age among mothers aged 15-49 years, by Demographic and Health Surveys round.** BFA, Burkina Faso; CI, confidence interval; CIV, Republic of Côte d’Ivoire; GHA, Republic of Ghana; JOR, Hashemite Kingdom of Jordan; KEN, Republic of Kenya; KMH, Kingdom of Cambodia; MOZ, Republic of Mozambique; PHL, Republic of the Philippines; SEN, Republic of Senegal; TZA, United Republic of Tanzania.

**
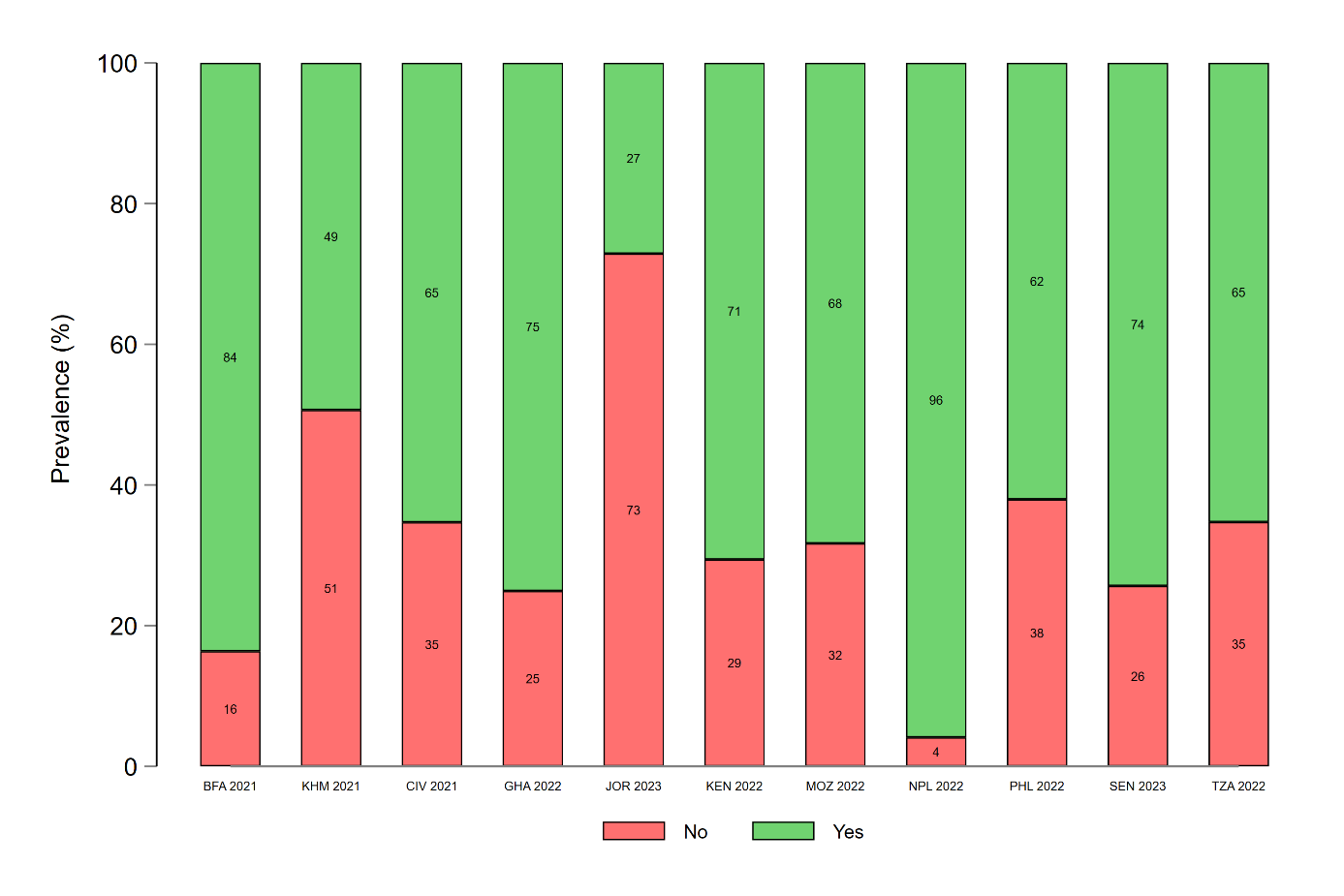
Supplemental figure 2. Percentage of infants and young children aged 12-23 months consuming breast milk, by Demographic and Healthy Survey round.** BFA, Burkina Faso; CIV, Republic of Côte d’Ivoire; GHA, Republic of Ghana; JOR, Hashemite Kingdom of Jordan; KEN, Republic of Kenya; KMH, Kingdom of Cambodia; MOZ, Republic of Mozambique; PHL, Republic of the Philippines; SEN, Republic of Senegal; TZA, United Republic of Tanzania.


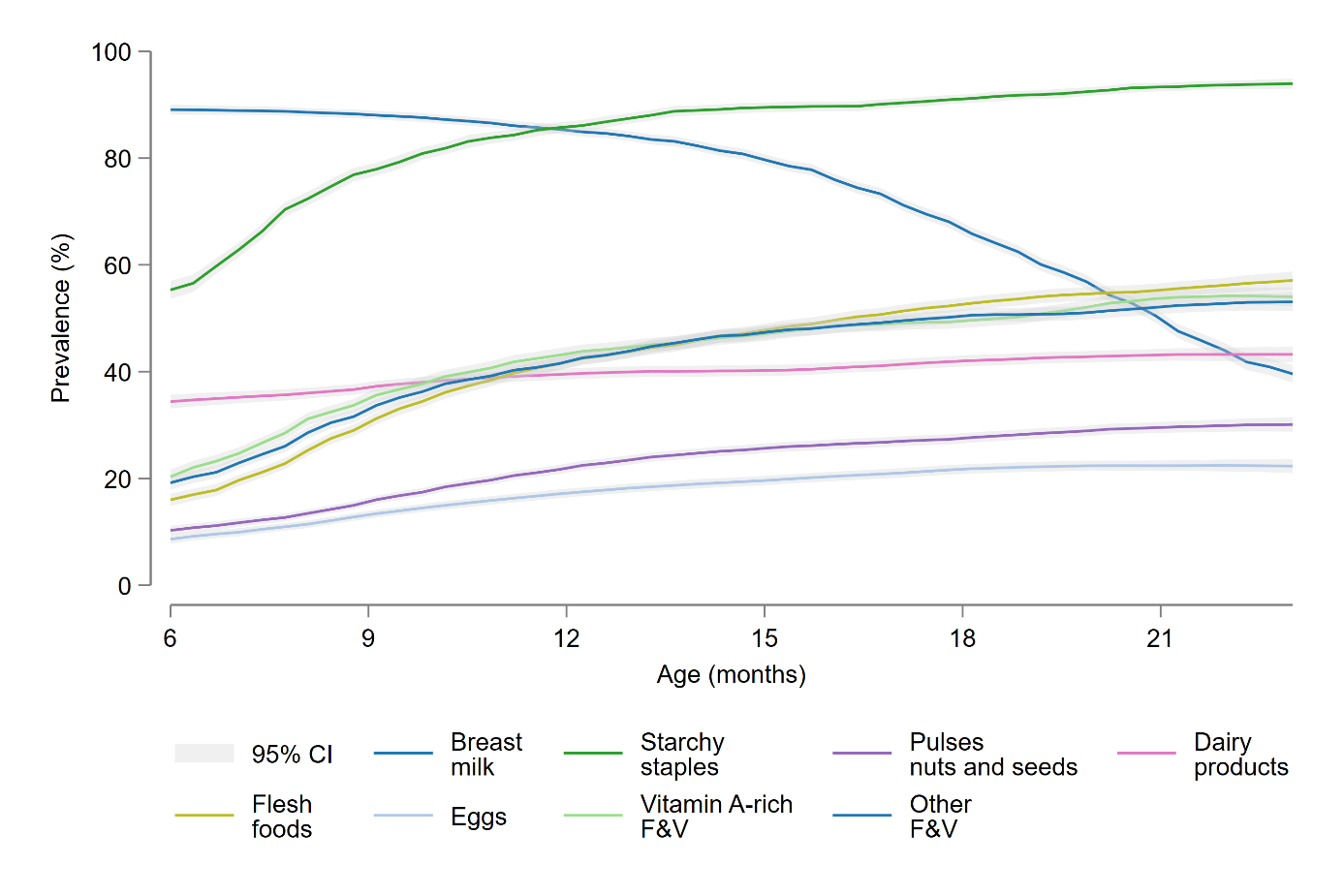
**Supplemental figure 3. Kernel-weighted local polynomial regression of nutritious food group consumption prevalence on age among infants and young children aged 6-23 months from 11 Demographic and Health Surveys round.** CI, confidence interval; F&V, fruits and vegetables.

**
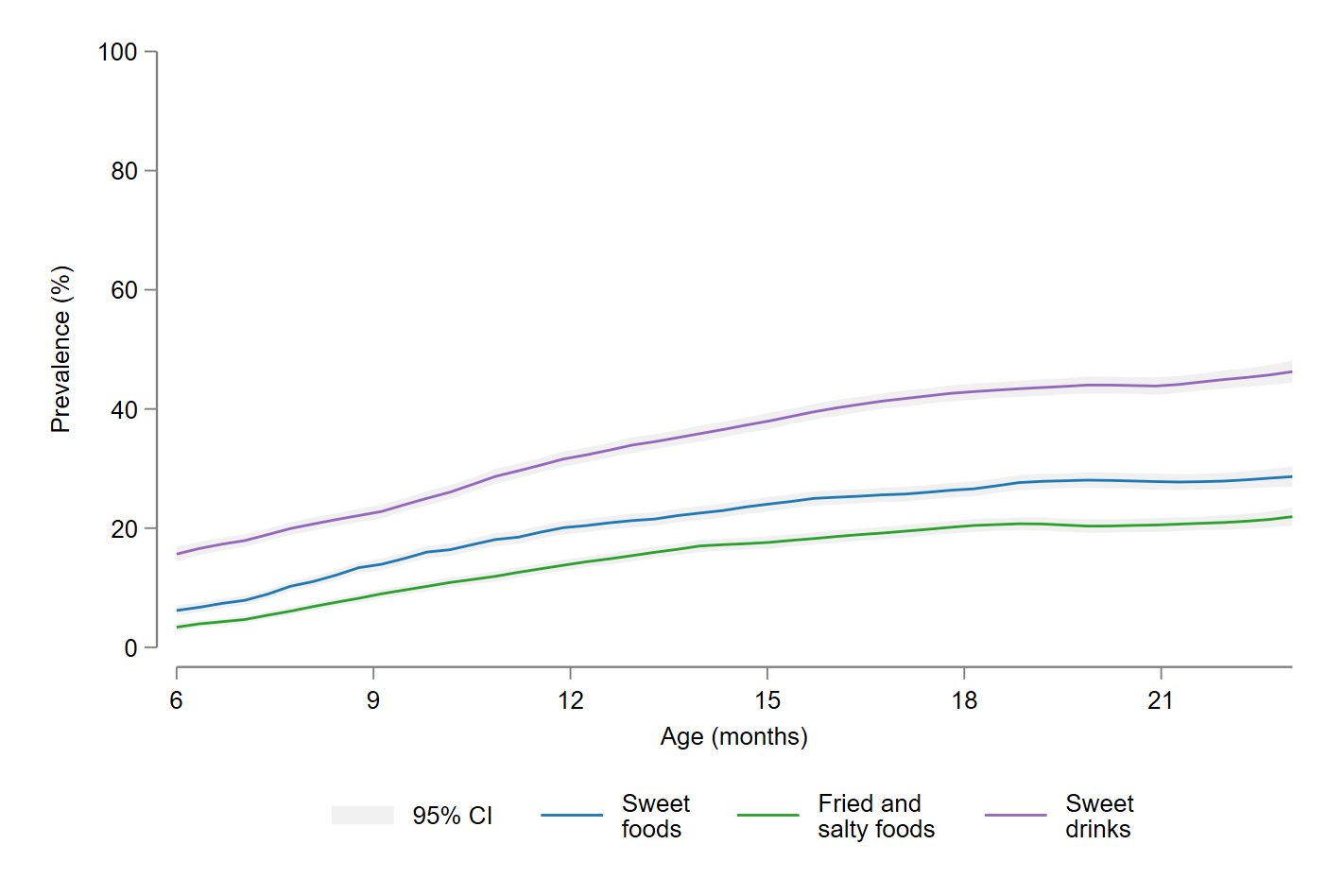
Supplemental figure 4. Kernel-weighted local polynomial regression of unhealthy food group consumption prevalence on age among infants and young children aged 6-23 months from 11 Demographic and Health Surveys (DHS) round.** CI, confidence interval.


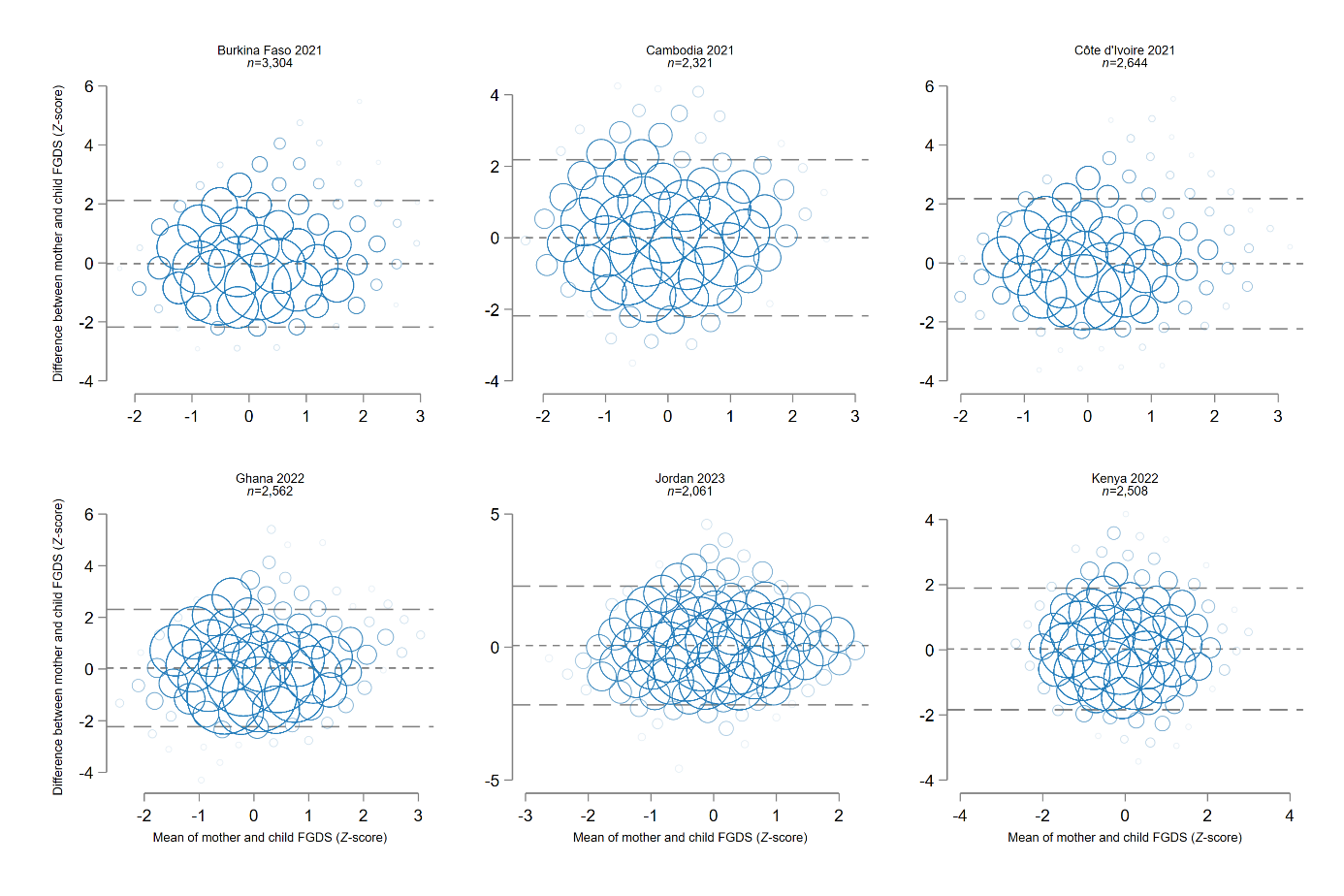
**Supplemental figure 5. Bland-Altman plots of weighted mean-standardized food group diversity score (FGDS) among infants and young children aged 6-23 months and their mothers aged 15-49 years, by Demographic and Healthy Survey round.** The short dashed line is the mean difference, whereas the 2 dashed lines are the upper and lower levels of agreement (mean ± 1.96 × standard deviation). The size of the bubbles is proportional to the number of data points.

**
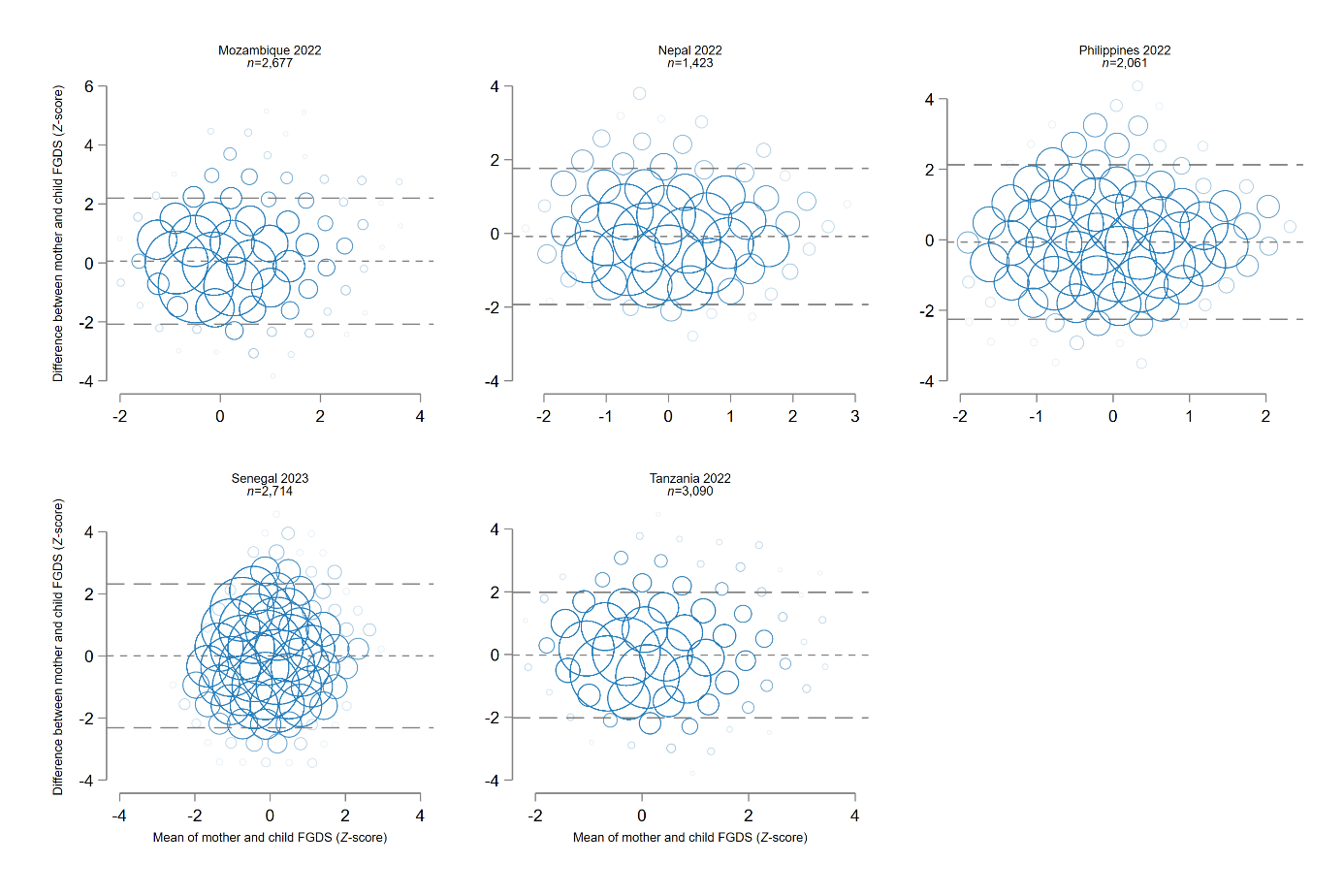
Supplemental figure 6. Bland-Altman plot of weighted mean-standardized food group diversity score (FGDS) among infants and young children aged 6-23 months and their mothers aged 15-49 years, by Demographic and Healthy Survey round.** The short dashed line is the mean difference, whereas the 2 dashed lines are the upper and lower levels of agreement (mean ± 1.96 × standard deviation). The size of the bubbles is proportional to the number of data points.

**
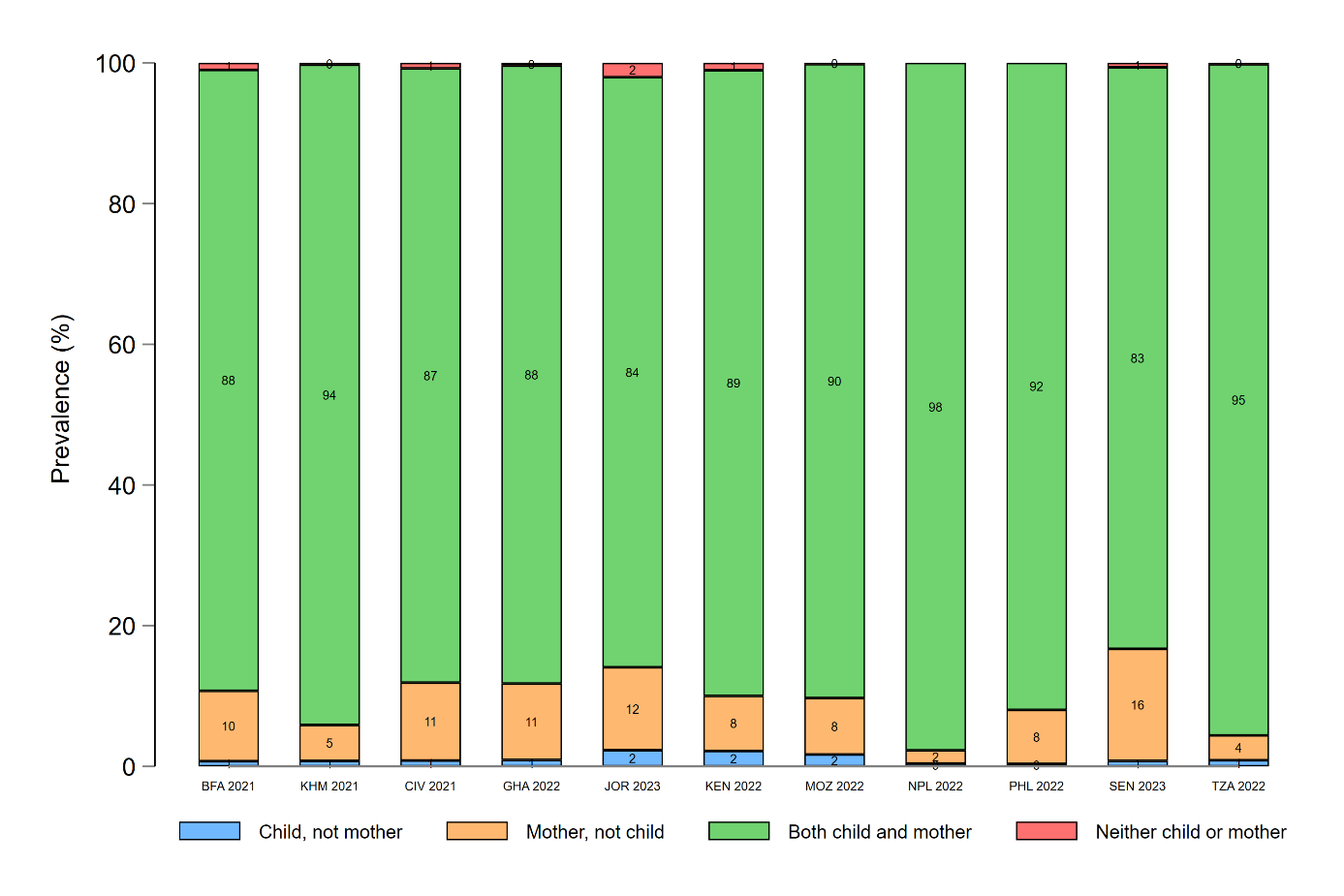
 Supplemental figure 7. Percentage concordance and discordance between starchy staples consumption among infants and young children aged 12-23 months and their mothers aged 15-49 years, by Demographic and Healthy Survey round.** The sum of the green and red bars is the prevalence of concordance, while the sum of the blue and orange bars is the prevalence of discordance. BFA, Burkina Faso; CIV, Republic of Côte d’Ivoire; GHA, Republic of Ghana; JOR, Hashemite Kingdom of Jordan; KEN, Republic of Kenya; KMH, Kingdom of Cambodia; MOZ, Republic of Mozambique; PHL, Republic of the Philippines; SEN, Republic of Senegal; TZA, United Republic of Tanzania.

**
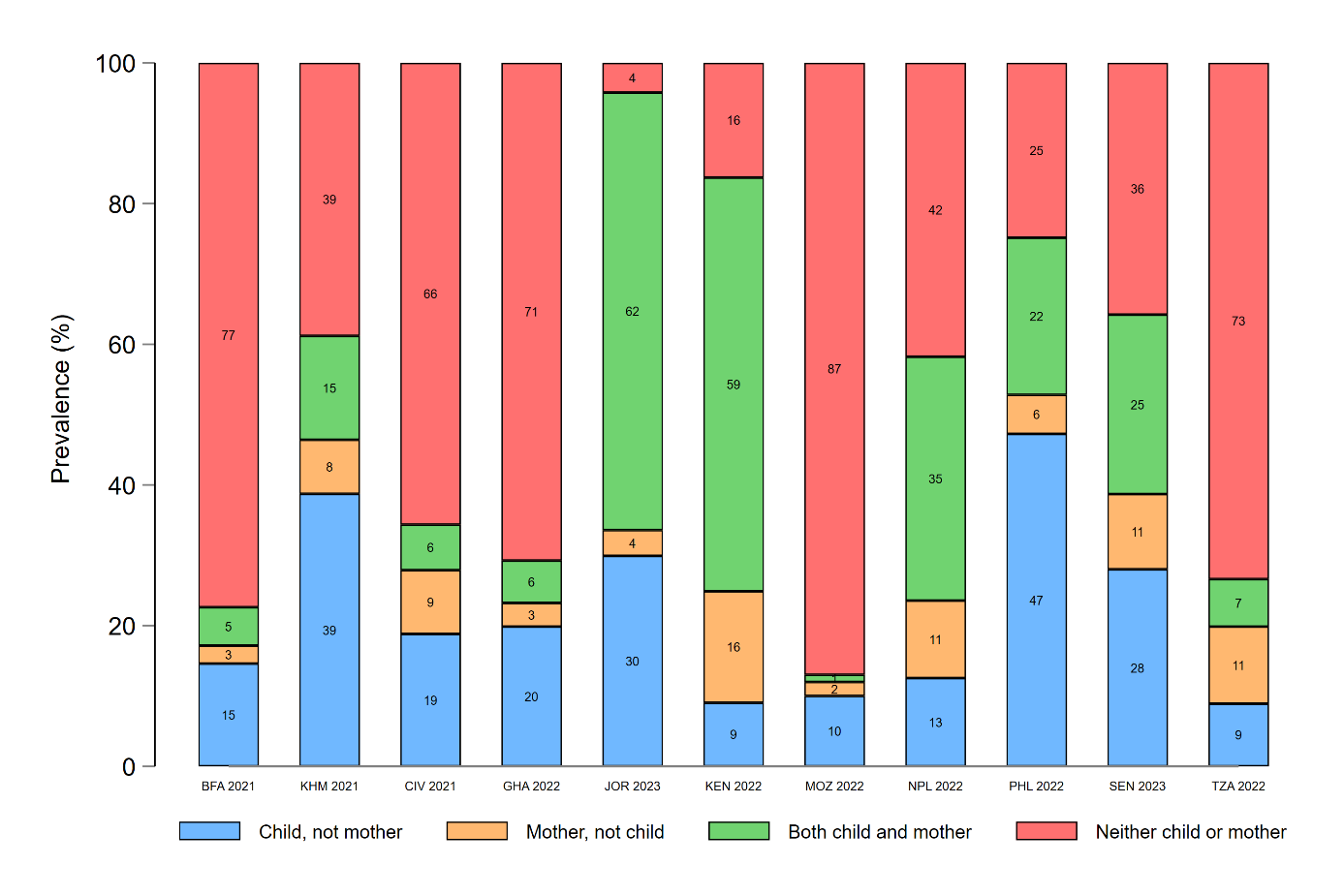
Supplemental figure 8. Percentage concordance and discordance between dairy product consumption among infants and young children aged 12-23 months and their mothers aged 15-49 years, by Demographic and Healthy Survey round.** The sum of the green and red bars is the prevalence of concordance, while the sum of the blue and orange bars is the prevalence of discordance. BFA, Burkina Faso; CIV, Republic of Côte d’Ivoire; GHA, Republic of Ghana; JOR, Hashemite Kingdom of Jordan; KEN, Republic of Kenya; KMH, Kingdom of Cambodia; MOZ, Republic of Mozambique; PHL, Republic of the Philippines; SEN, Republic of Senegal; TZA, United Republic of Tanzania.

**
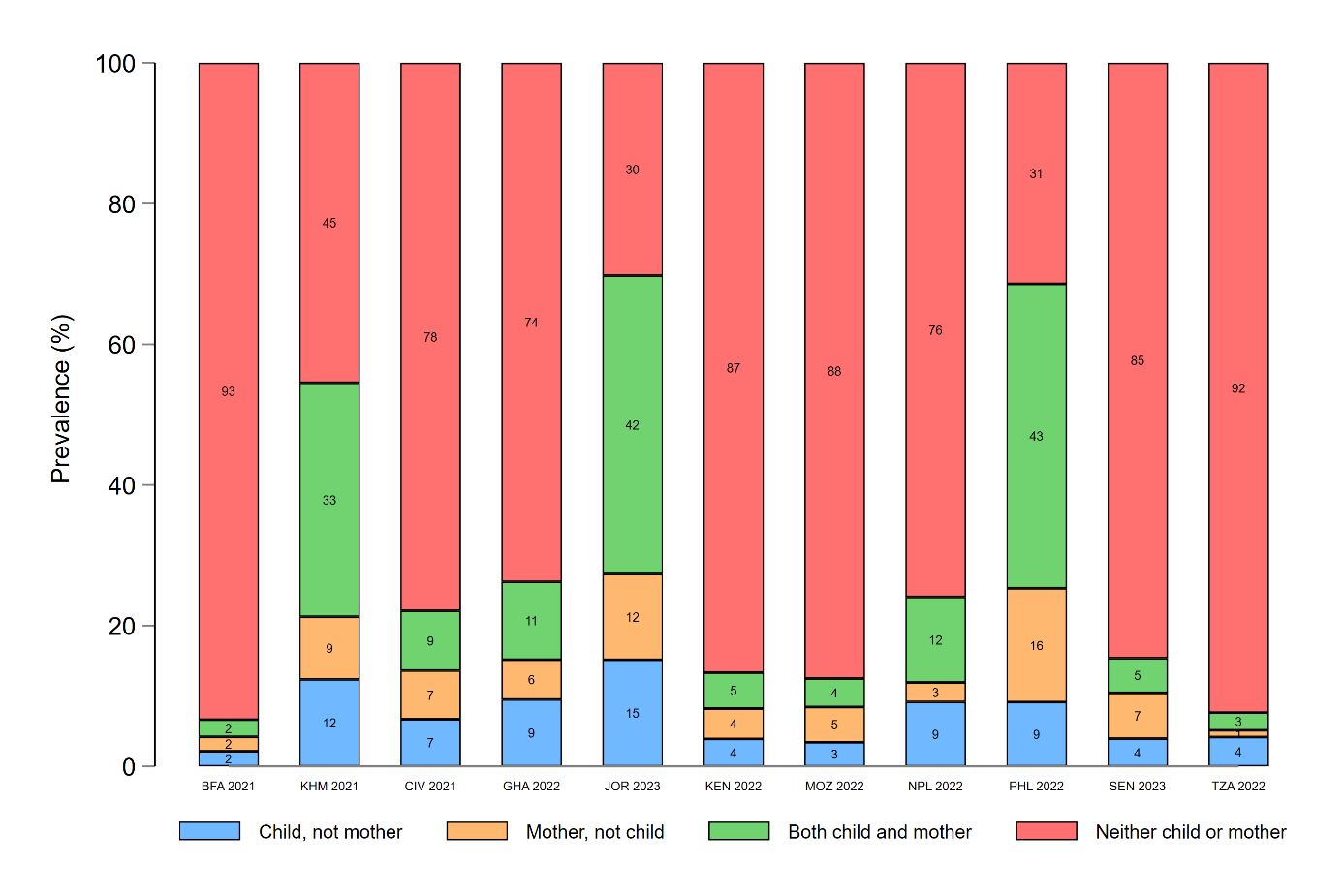
Supplemental figure 9. Percentage concordance and discordance between eggs consumption among infants and young children aged 12-23 months and their mothers aged 15-49 years, by Demographic and Healthy Survey round.** The sum of the green and red bars is the prevalence of concordance, while the sum of the blue and orange bars is the prevalence of discordance. BFA, Burkina Faso; CIV, Republic of Côte d’Ivoire; GHA, Republic of Ghana; JOR, Hashemite Kingdom of Jordan; KEN, Republic of Kenya; KMH, Kingdom of Cambodia; MOZ, Republic of Mozambique; PHL, Republic of the Philippines; SEN, Republic of Senegal; TZA, United Republic of Tanzania.


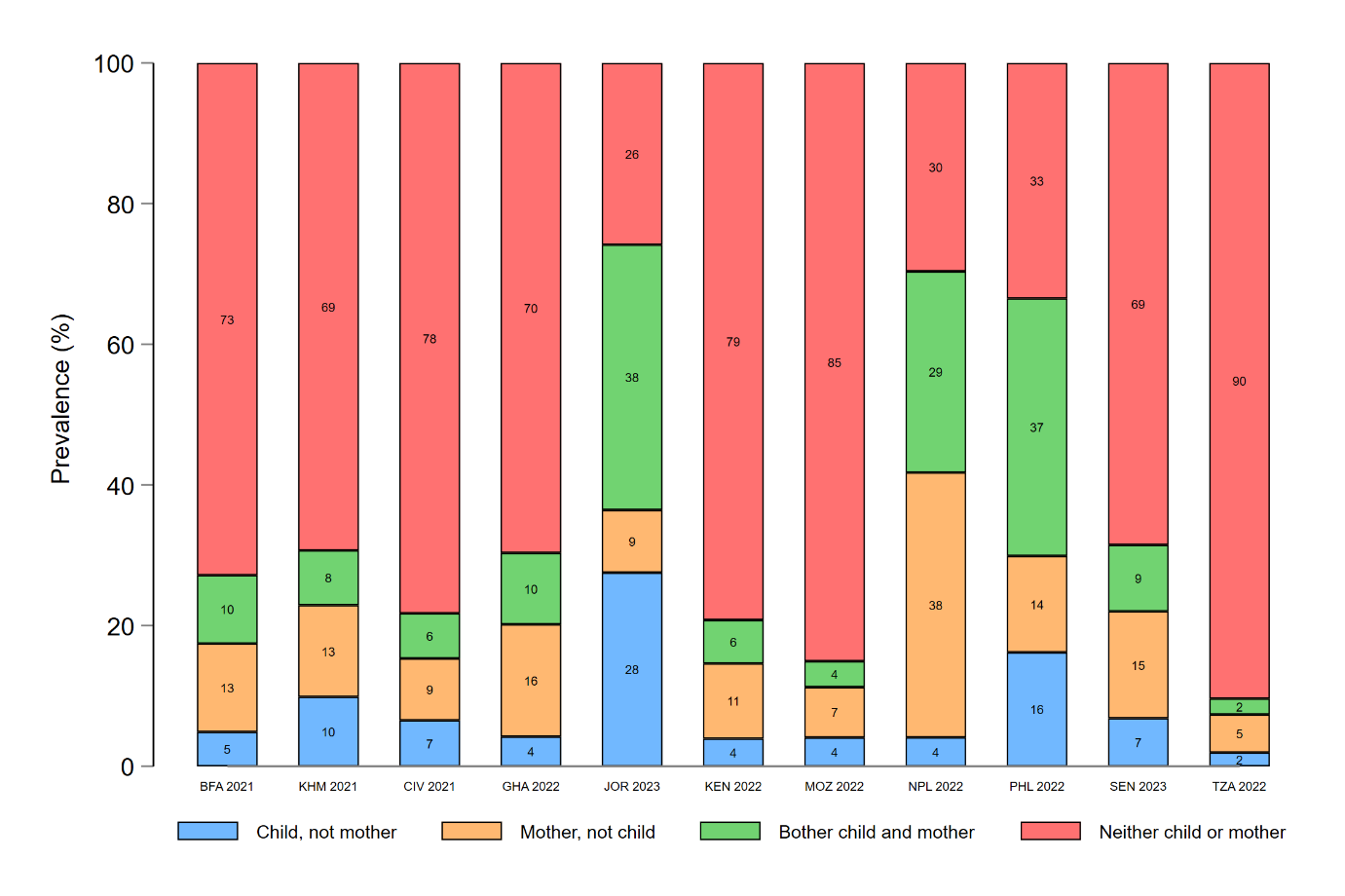


**Supplemental figure 10. Percentage concordance and discordance between sweet foods consumption among infants and young children aged 12-23 months and their mothers aged 15-49 years, by Demographic and Healthy Survey round.** The sum of the green and red bars is the prevalence of concordance, while the sum of the blue and orange bars is the prevalence of discordance. BFA, Burkina Faso; CIV, Republic of Côte d’Ivoire; GHA, Republic of Ghana; JOR, Hashemite Kingdom of Jordan; KEN, Republic of Kenya; KMH, Kingdom of Cambodia; MOZ, Republic of Mozambique; PHL, Republic of the Philippines; SEN, Republic of Senegal; TZA, United Republic of Tanzania.


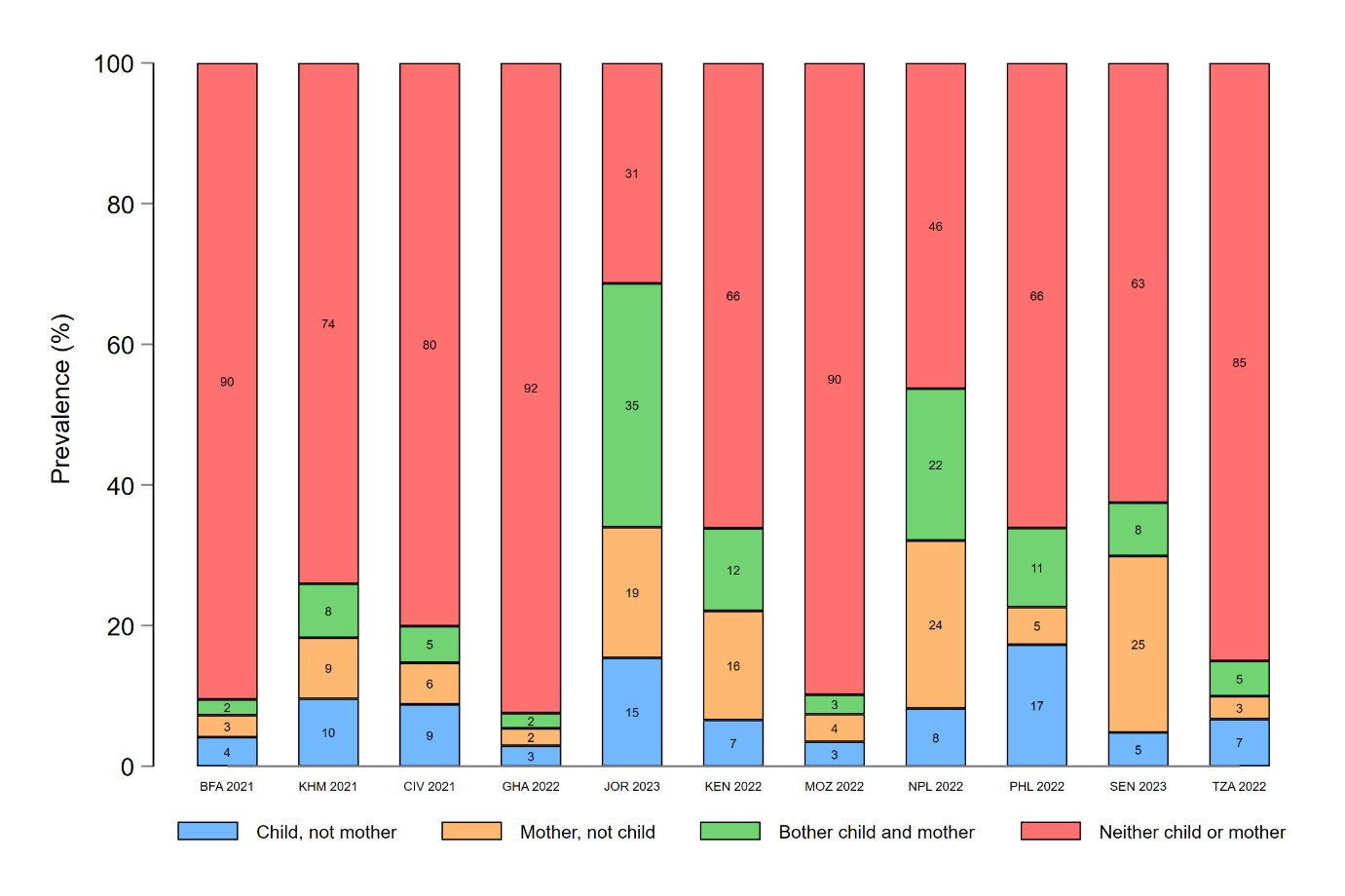
**Supplemental figure 11. Percentage concordance and discordance between fried and salty foods consumption among infants and young children aged 12-23 months and their mothers aged 15-49 years, by Demographic and Healthy Survey round.** The sum of the green and red bars is the prevalence of concordance, while the sum of the blue and orange bars is the prevalence of discordance. BFA, Burkina Faso; CIV, Republic of Côte d’Ivoire; GHA, Republic of Ghana; JOR, Hashemite Kingdom of Jordan; KEN, Republic of Kenya; KMH, Kingdom of Cambodia; MOZ, Republic of Mozambique; PHL, Republic of the Philippines; SEN, Republic of Senegal; TZA, United Republic of Tanzania.


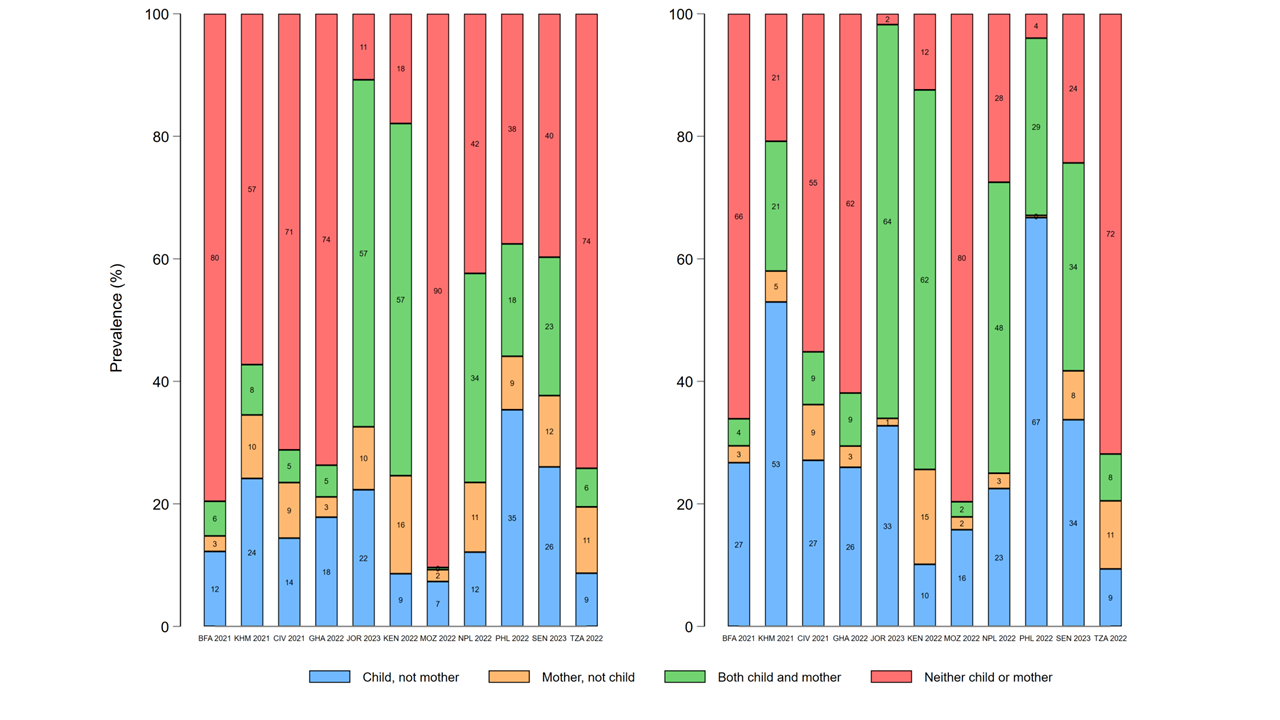
**Supplemental figure 12. Percentage concordance and discordance between dairy products consumption among breastfed and non-breastfed infants and young children aged 12-23 months and their mothers aged 15-49 years, by Demographic and Healthy Survey round.** The sum of the green and red bars is the prevalence of concordance, while the sum of the blue and orange bars is the prevalence of discordance. BFA, Burkina Faso; CIV, Republic of Côte d’Ivoire; GHA, Republic of Ghana; JOR, Hashemite Kingdom of Jordan; KEN, Republic of Kenya; KMH, Kingdom of Cambodia; MOZ, Republic of Mozambique; PHL, Republic of the Philippines; SEN, Republic of Senegal; TZA, United Republic of Tanzania.

**
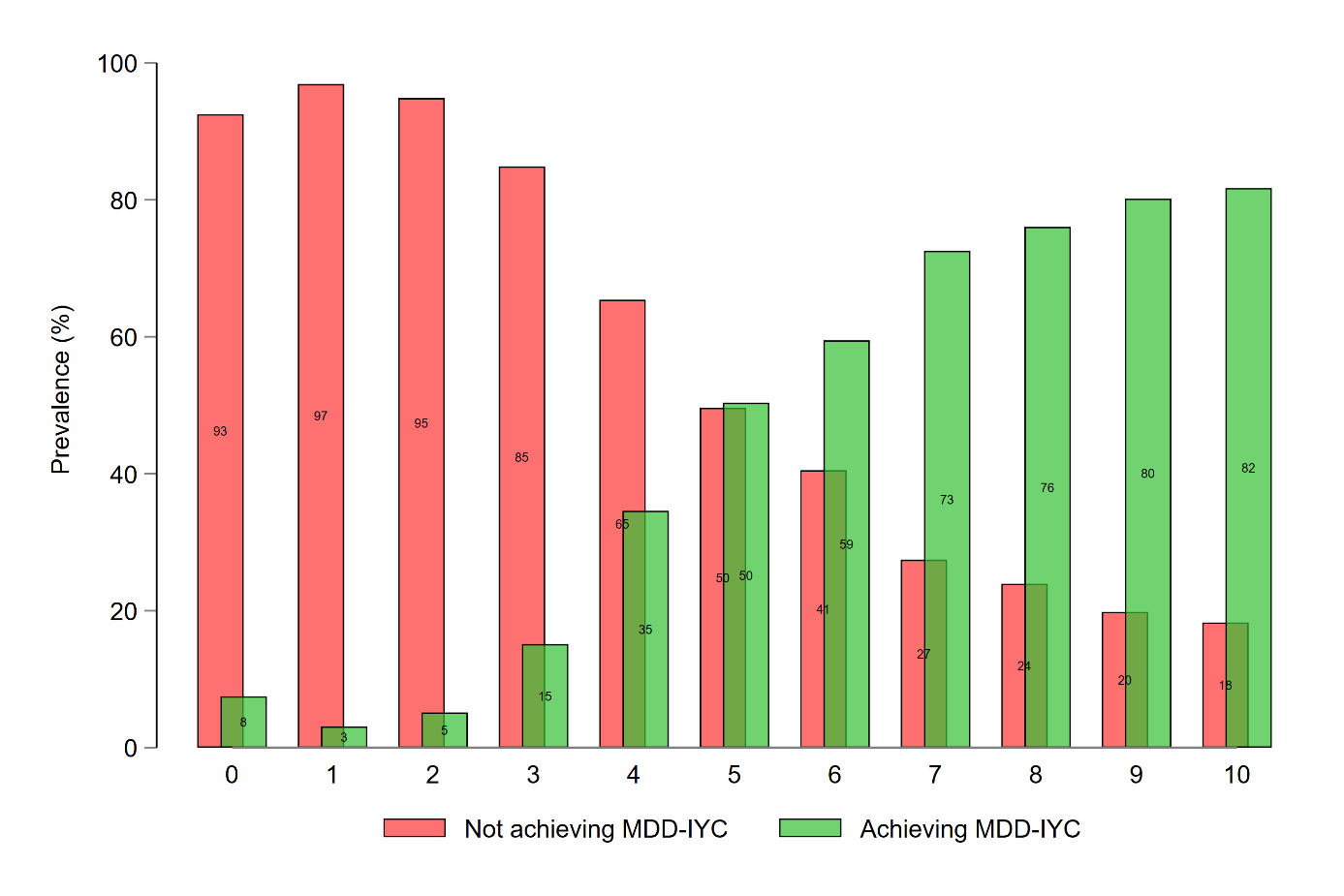
Supplemental figure 13. Percentage of infants and young children aged 12-23 months from 11 Demographic and Healthy Survey rounds (*n*=18,770) achieving Minimum Dietary Diversity for Infants and Young Children (MDD-IYC), by maternal food group diversity score (0-10 points)**

**
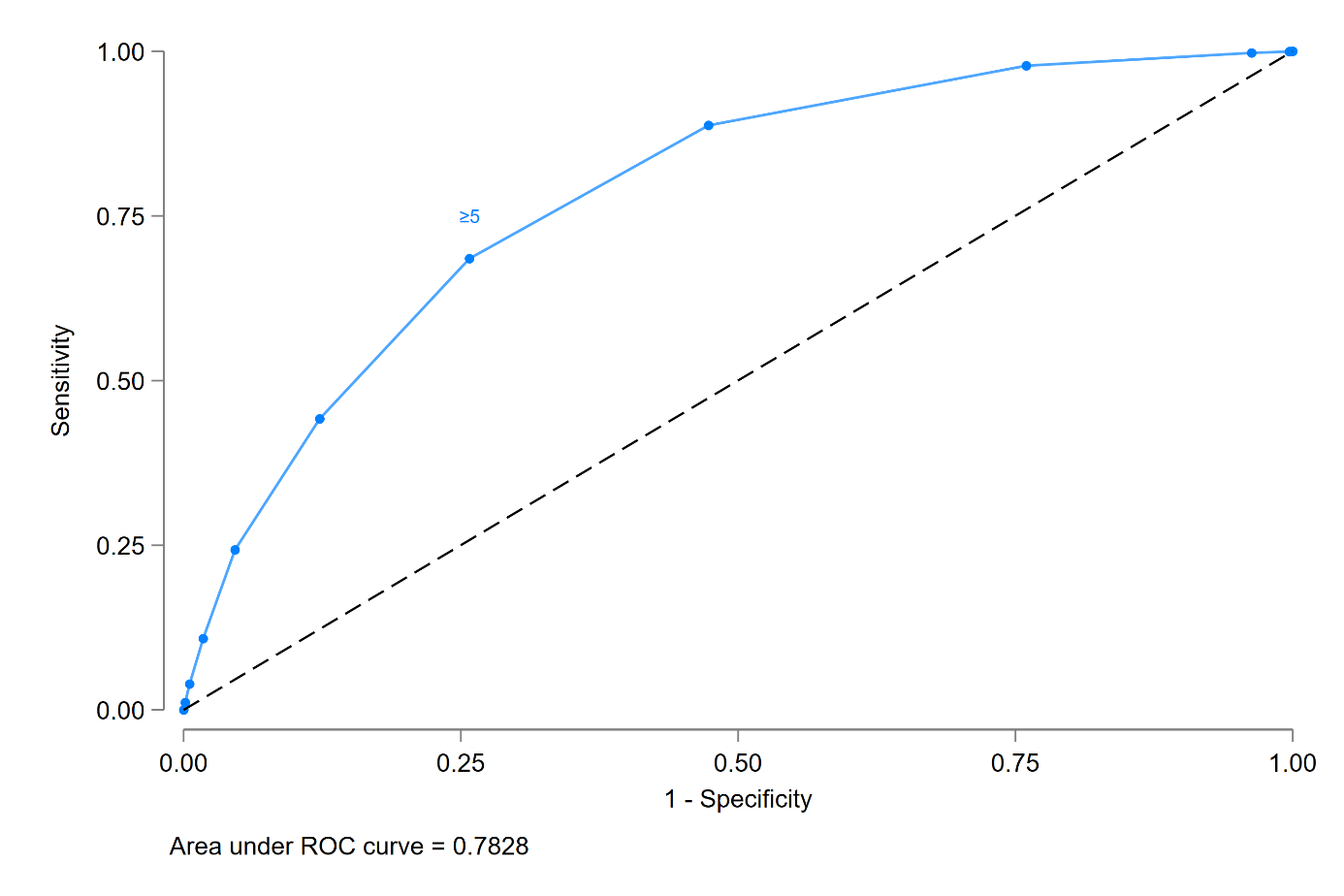
 Supplemental figure 14. Receiver operating characteristic (ROC) curve of maternal food group diversity score (0-10 points) indicating predictions for Minimum Dietary Diversity for Infants and Young Children (MDD-IYC) (12-23 months) from 11 Demographic and Healthy Survey rounds (*n*=18,770).** In the pooled sample, ≥5 food groups or Minimum Dietary Diversity for Women (MDD-W) showed the best balance between sensitivity (68.5%), specificity (74.2%), and percentage correctly classified (72.2%).
